# Accelerometer-derived rest-activity rhythms, genetic risk, and chronic inflammatory markers and the risk of abdominal aortic aneurysm

**DOI:** 10.3389/fpubh.2026.1800800

**Published:** 2026-04-30

**Authors:** Yongliang Zhong, Xinyi Liu, Zhiyu Qiao, Haiou Hu, Chengnan Li, Yipeng Ge, Junming Zhu

**Affiliations:** Department of Cardiovascular Surgery, Beijing Aortic Disease Center, Beijing Anzhen Hospital of Capital Medical University, Beijing, China

**Keywords:** abdominal aortic aneurysm, accelerometry, inflammation, rest-activity rhythm, XGBoost

## Abstract

**Introduction:**

Disrupted rest-activity rhythm (RAR), an accelerometer-derived measure of the strength and temporal organization of daily behavioral rhythms, has been linked to multiple adverse health outcomes. Given that the development of abdominal aortic aneurysm (AAA) involves chronic inflammation, extracellular matrix degradation, and vascular smooth muscle cell dysfunction, attenuated rest-activity rhythms (RAR) may be associated with AAA risk. However, prospective evidence on this association remains limited.

**Methods:**

The primary analysis included 78,282 UK Biobank participants who completed accelerometer monitoring between 2013 and 2015. Thirteen parametric and nonparametric RAR parameters were derived. Cox proportional hazards models estimated associations between RAR parameters and AAA incidence, as well as potential interactions and joint effects with other risk factors. Causal mediation analysis examined the mediating role of inflammatory markers. An XGBoost-based survival model evaluated variable importance, and SHapley Additive exPlanations (SHAP) interpreted feature contributions.

**Results:**

During a mean follow-up of 10.1 years, 229 AAA cases were recorded. Lower RAR parameters, particularly relative amplitude (RA), M10, amplitude, and mesor, were significantly associated with higher AAA risk [lowest vs. highest tertile HRs: RA 1.49 (95% CI 1.03–2.15); M10 1.51 (1.05–2.18); amplitude 1.58 (1.09–2.30); mesor 1.46 (1.04–2.14)]. Although no significant interactions were observed, individuals with weaker RAR combined with current smoking or high polygenic risk had markedly increased AAA risk. Mediation analysis indicated that neutrophil and monocyte counts explained about 5–6% of the RAR-AAA association. In the XGBoost model, beyond age, male sex, and PRS as dominant predictors, mesor, RA, and M10 emerged as meaningful contributors to AAA risk prediction.

**Conclusion:**

Accelerometer-derived RAR parameters are strongly associated with the risk of developing AAA. Integrating RAR measures with genetic and traditional risk factors may improve risk stratification and provide novel insights for preventive strategies against AAA.

## 1 Introduction

Abdominal aortic aneurysm (AAA) is a highly lethal vascular disorder characterized by a localized dilatation of the abdominal aorta (≥50% increase in diameter or ≥30 mm) that is prone to rupture, leading to extremely high mortality (1). Despite advances in imaging and surgical repair techniques, the prevention and early detection of AAA remain limited due to the lack of modifiable behavioral risk markers (2). Although traditional risk factors, such as older age, male sex, smoking, and genetic susceptibility, are well established (3), emerging evidence suggests that disruption of circadian and behavioral rhythms may also contribute to vascular pathophysiology and increase the risk of AAA development (4).

The rest-activity rhythm (RAR), derived from wrist-worn accelerometers, provides an objective measure of circadian patterns in daily behavior. Beyond serving as a proxy for the sleep–wake cycle, RAR captures multidimensional features of biological rhythmicity, including amplitude, stability, and fragmentation (5). Recent large-scale cohort studies have demonstrated that disrupted RAR patterns, such as reduced amplitude, irregular timing, or weakened rhythmicity, are strongly associated with adverse health outcomes, including all-cause mortality, cardiovascular disease, cancer, and metabolic disorders (6, 7). Mechanistically, circadian disruption may promote endothelial dysfunction, chronic inflammation, and extracellular matrix degradation-key processes in the development of AAA (8). However, no prospective study to date has evaluated whether disturbed RAR patterns are associated with future AAA risk in the general population.

Given that both circadian misalignment and physical inactivity can trigger systemic inflammation and metabolic stress (9), the potential interplay among RAR, inflammation, and genetic susceptibility warrants further investigation. Moreover, polygenic risk scores (PRS) have recently emerged as reliable predictors of AAA risk (10), yet it remains unclear whether behavioral rhythms can modify genetic susceptibility. Understanding these multidimensional interactions may provide new behavioral insights into the pathogenesis of AAA.

Therefore, using accelerometer data from the UK Biobank, we comprehensively examined the associations of both parametric and nonparametric RAR parameters with AAA incidence. We further explored the potential modifying and mediating effects of genetic risk and inflammatory markers, and finally applied machine learning models to assess the relative importance of different RAR parameters in predicting AAA occurrence.

## 2 Methods

### 2.1 Study design and participants

This study utilized data from the UK Biobank, a large-scale, population-based prospective cohort including over half a million participants. Between 2006 and 2010, individuals aged 37–73 years were recruited from 22 assessment centers throughout the United Kingdom (11). At enrollment, participants completed an extensive touchscreen questionnaire capturing information on sociodemographic characteristics, lifestyle factors, and medical history. Comprehensive descriptions of the study design and data collection procedures have been reported elsewhere. The UK Biobank received ethical approval from the North West Multi-Centre Research Ethics Committee (Ref: 11/NW/0382) (see https://www.ukbiobank.ac.uk/learn-more-about-uk-biobank/about-us/ethics).

Between May 2013 and December 2015, a total of 106,053 participants were invited to wear an accelerometer for 7 consecutive days (12). Among them, 103,660 participants provided sufficient recordings to derive acceleration data aggregated in 5-s epochs. We further excluded participants with data quality issues (e.g., recording errors, daylight saving time crossover, calibration problems, or interrupted recording periods), those with fewer than three valid days of data (a valid day was defined as having complete epoch data after imputation, with <4 h of imputed data), participants of non-White British ancestry, those with a baseline diagnosis of AAA, and those missing key baseline information. After these exclusions, a total of 78,282 participants were included in the final analysis. Supplementary Figure S1 presents a flowchart summarizing this selection process.

### 2.2 Rest-activity rhythm

Accelerometer data were collected using a wrist-worn Axivity AX3 triaxial accelerometer. Participants were instructed to wear the device on their dominant wrist continuously for seven consecutive days. Data were processed according to previously described methods (12). Briefly, raw acceleration signals were calibrated to local gravity and recorded at a frequency of 100 Hz within a dynamic range of ±8 g. The Euclidean norm of acceleration across all three axes was calculated, with gravitational acceleration and device noise removed, to derive the vector magnitude for each 5-s epoch. To reduce noise, the 5-s data were further aggregated into 1-min time series. Based on these 1-min data, thirteen rest–activity rhythm parameters were derived using both parametric and nonparametric approaches, as established in prior studies (13). The parametric approach was based on the extended cosine model, which included the pseudo-F statistic, amplitude, midline estimating statistic of rhythm (mesor), upper mesor, lower mesor, and acrophase (peak time). The nonparametric approach yielded seven additional parameters: interdaily stability (IS), intradaily variability (IV), the least active 5-h period (L5), the most active 10-h period (M10), relative amplitude (RA), L5 start time, and M10 start time. Detailed descriptions of all rest-activity rhythm parameters are provided in Supplementary Table S1. Because the midpoint of L5 spanned midnight, this variable was expressed as hours from midnight to avoid discontinuity in its values. All rhythm parameters were categorized into tertiles, with the tertile corresponding to the lowest risk selected as the reference group, specifically Tertile 1 for IV, L5, and L5 start time; Tertile 2 for upper mesor, lower mesor, acrophase, and M10 start time; and Tertile 3 for the remaining seven parameters.

### 2.3 Accelerometer-derived activity patterns

To examine the associations between daily physical activity duration and RAR, we further utilized a previously validated machine-learning algorithm to accurately classify accelerometer data into light physical activity (LPA), moderate-to-vigorous physical activity (MVPA), sedentary behavior, and sleep (14). For MVPA, participants were categorized according to the World Health Organization (WHO) physical activity guidelines into two groups: an inactive group (MVPA < 150 min/week) and an active group (MVPA ≥ 150 min/week). In addition, based on the temporal distribution of MVPA, participants were further divided into three groups: an inactive group (MVPA < 150 min/week), a regularly active group (with at least 50% of total MVPA accumulated over more than two days per week), and a weekend warrior group (with at least 50% of total MVPA accumulated within one or two days per week).

### 2.4 Polygenic risk score calculation

The PRS for AAA was derived using PRS-CS, a Bayesian regression approach that applies continuous shrinkage priors to model genome-wide effect sizes (15). GWAS summary statistics were obtained from the largest available study of AAA conducted in populations of European ancestry, as reported in the GWAS Catalog. PRS-CS incorporates external linkage disequilibrium (LD) patterns from the 1,000 Genomes Project European reference panel to estimate posterior SNP effect sizes while accounting for polygenicity and inter-variant correlations (16). The global shrinkage parameter was fixed at the default setting recommended by the method’s developers. For each participant, individual PRS values were computed as the sum of the estimated posterior effect sizes across all SNPs. According to the distribution of PRS values, participants were further categorized into three genetic risk groups: low (lowest tertile), intermediate (middle tertile), and high (highest tertile).

### 2.5 Covariate assessment

Covariates included demographic characteristics, baseline medical history, lifestyle factors, and chronic inflammatory markers. Demographic characteristics comprised age, sex, educational level, Townsend deprivation index, employment status, body mass index (BMI), and shift work. Baseline medical history included the presence of chronic respiratory disease, chronic liver disease, chronic kidney disease, other cardiovascular diseases, hypertension, diabetes, and dyslipidemia. Lifestyle factors included smoking status (never, former, or current) and alcohol consumption, with men consuming more than two standard drinks per day and women consuming more than one defined as drinkers, based on self-reported information. Criteria for a healthy diet are described in detail in Supplementary Table S2.

In the UK Biobank, peripheral blood cell counts were measured using a clinically validated Coulter LH 750 automated hematology analyzer, with quality control procedures conducted according to the manufacturer’s recommendations. Baseline measurements of neutrophil, monocyte, platelet, and lymphocyte counts were obtained. Based on these values, two systemic inflammatory indices were calculated to reflect immune–inflammatory status: the systemic inflammation response index (SIRI), defined as neutrophil count × monocyte count / lymphocyte count, and the systemic immune–inflammation index (SII), defined as neutrophil count × platelet count / lymphocyte count.

### 2.6 Outcomes

The primary outcome was the occurrence of AAA, identified using ICD-10 codes I71.3 and I71.4 from death registry data, primary care records, hospital admissions, or self-reported diagnoses. Incident cases were defined as the earliest recorded diagnosis of AAA. Follow-up time was calculated from the end date of accelerometer wear to the date of AAA diagnosis, death, loss to follow-up, or end of the study period, whichever occurred first.

### 2.7 Statistical analysis

Baseline characteristics were summarized as means (standard deviations) for continuous variables and counts (percentages) for categorical variables. Differences across groups were assessed using one-way analysis of variance for continuous variables and the chi-square test for categorical variables. Cox proportional hazards models were used to estimate hazard ratios (HRs) and 95% confidence intervals (CIs), with the proportional hazards assumption evaluated using Schoenfeld residuals. Two sequential models were constructed: Model 1, adjusted for age and sex; and Model 2, further adjusted for education, employment status, Townsend deprivation index, shift work, body mass index (BMI), medical history, smoking status, diet, alcohol consumption, and statin/vitamin use. Before fitting Model 2, we evaluated multicollinearity among the covariates using variance inflation factors. The results indicated no substantial multicollinearity among the included variables (Supplementary Table S3). To address multiple testing, we applied the Benjamini-Hochberg procedure to control the false discovery rate (FDR) for all *p* values across the 13 rest activity rhythm variables in Model 1 and Model 2 separately. This approach was chosen because these variables reflect correlated dimensions of the same underlying rest activity rhythm construct, and a family wise error rate approach would likely be overly conservative. This correction was performed across the 13 circadian rhythm related variables, separately for Model 1 and Model 2. Restricted cubic spline (RCS) regression was applied to examine potential nonlinear associations between significant rest–activity rhythm parameters and AAA incidence identified in the Cox models. Stratified analyses were further performed by age, sex, and BMI categories (18.5–24.9 vs. outside this range).

Multiple sensitivity analyses were conducted to test the robustness of the results. To minimize potential reverse causality, participants diagnosed with AAA within the first year of follow-up were excluded. To account for associations between rest–activity rhythm and different activity types, additional adjustments were made for time spent in four activity categories. To account for potential genetic confounding, analyses were further adjusted for the PRS. Fine–Gray subdistribution hazard models were used to account for competing risks from non-AAA deaths or loss to follow-up. Missing covariate data were assumed to be missing at random conditional on the observed data and were therefore handled using multiple imputation.

To explore the potential associations of rest–activity rhythm parameters and multiple covariates with AAA risk, Cox proportional hazards models were first applied to evaluate the relationships of different activity types, MVPA categories, and PRS categories with AAA incidence. We then assessed the interactions between rest–activity rhythm parameters that were significantly associated with AAA and various covariates. Multiplicative interactions were examined using cross-product terms, while additive interactions were evaluated by calculating the relative excess risk due to interaction (RERI) and corresponding 95% CIs. For joint effect analyses, the reference group was defined as the category associated with the lowest AAA risk for each variable.

Additionally, exploratory mediation analysis was performed to examine the potential mediating effects of multiple inflammatory markers in the association between rest–activity rhythm and AAA risk, adjusting for covariates included in Model 2. The 95% CIs for the proportion mediated were estimated using 1,000 quasi-Bayesian Monte Carlo simulations with bootstrap resampling.

To evaluate the relative importance of RAR variables in predicting incident AAA, we developed four survival models using the extreme gradient boosting (XGBoost) algorithm: (1) a model including baseline covariates only; (2) baseline covariates plus PRS; (3) baseline covariates plus RAR; and (4) baseline covariates plus PRS plus RAR. The dataset was randomly split into a training set (70%) and a validation set (30%). In the training set, the optimal number of boosting iterations was determined using early stopping. Features included demographic characteristics, socioeconomic factors, lifestyle behaviors, baseline health status, medication and supplement use, and physical activity related variables. Model performance was evaluated in the validation set. Discrimination was assessed using the area under the receiver operating characteristic curve (AUC). To evaluate overall predictive accuracy and calibration, we calculated the Brier score and generated calibration plots. Clinical utility was assessed using decision curve analysis, and internal validation was performed using five-fold cross validation. Finally, to quantify the contribution of each variable to AAA risk prediction in Model 4, we used SHapley additive explanations (SHAP) to interpret the XGBoost model. SHAP values represent the marginal contribution of each variable to the model output (log hazard), with positive values indicating higher risk and negative values indicating lower risk. For each feature, the mean absolute SHAP value (mean |SHAP|) was calculated across all individuals in the validation set as an overall measure of importance. The “shapviz” package was used to generate SHAP beeswarm plots to visualize the distribution of feature impacts, directionality, and potential non-linear patterns.

All statistical analyses were conducted using R software (version 4.4.1; R Foundation for Statistical Computing, Vienna, Austria). All tests were two-sided, and *p* < 0.05 was considered statistically significant.

## 3 Results

### 3.1 Baseline characteristics of participants

Baseline characteristics of the participants are presented in Table 1. A total of 78,282 participants were included in the analysis, with a mean age of 56.3 years (SD = 7.8), and 43.9% were men. When categorized by tertiles of the pseudo-F statistic, participants in the lowest tertile, indicating weaker overall rhythmicity, were more likely to be younger, male, engaged in any shift work, and current or former smokers.

**Table 1:** Baseline characteristics of participants according to tertiles of pseudo-F statistic.

Characteristics	Pseudo-F statistic			*P*-value
	Tertile 1	Tertile 2	Tertile 3	
*N*	26,094	26,094	26,094	
Pseudo-F statistic (range)	48.1 ± 15.8	90.5 ± 11.1	147.5 ± 34.5	
Demographics				
Age (years)	53.7 ± 8.1	57.1 ± 7.5	58.1 ± 7.1	< 0.001
Male (%)	14,385 (55.1%)	11,019 (42.2%)	8,932 (34.2%)	< 0.001
Townsend deprivation index	−1.5 ± 2.9	−1.9 ± 2.7	−2.1 ± 2.6	< 0.001
University or college degree (%)	13,740 (52.7%)	11,345 (43.5%)	9,765 (37.4%)	< 0.001
Employed, student, or retired (%)	24,337 (93.3%)	24,074 (92.3%)	23,801 (91.2%)	< 0.001
Shift work (%)	2,533 (9.71%)	2059 (7.89%)	2013 (7.71%)	< 0.001
BMI	26.8 ± 4.7	26.9 ± 4.5	26.5 ± 4.1	< 0.001
Lifestyle				
Smoking status (%)				< 0.001
Never	10,282 (39.4%)	10,271 (39.4%)	10,326 (39.6%)	
Previous	13,960 (53.5%)	14,170 (54.3%)	14,158 (54.3%)	
Current	1852 (7.10%)	1,653 (6.33%)	1,610 (6.17%)	
No heavy alcohol (%)	12,355 (47.3%)	11,876 (45.5%)	11,028 (42.3%)	< 0.001
Healthy diet (%)	2,618 (10.0%)	2,501 (9.58%)	2,727 (10.5%)	0.004
LPA time (day, min)	257.0 ± 88.1	297.0 ± 85.7	356.0 ± 91.4	< 0.001
MVPA time (day, min)	40.3 ± 30.8	38.4 ± 31.7	46.0 ± 40.4	< 0.001
Sedentary time (day, min)	620.0 ± 103.1	570.1 ± 93.3	502.1 ± 93.1	< 0.001
Sleep time (day, min)	523.0 ± 82.2	535.0 ± 72.2	537.0 ± 64.7	< 0.001
Medical history				
Hypertension (%)	6,483 (24.8%)	7,632 (29.2%)	7,338 (28.1%)	< 0.001
Diabetes (%)	1,404 (5.38%)	1,470 (5.63%)	1,365 (5.23%)	0.122
Dyslipidemia (%)	9,031 (34.6%)	9,467 (36.3%)	9,022 (34.6%)	< 0.001
Chronic respiratory diseases (%)	3,426 (13.1%)	3,373 (12.9%)	3,347 (12.8%)	0.577
Chronic liver disease (%)	69 (0.26%)	64 (0.25%)	68 (0.26%)	0.901
Chronic kidney disease (%)	102 (0.39%)	70 (0.27%)	57 (0.22%)	< 0.001
Cardiovascular disease (%)	2,850 (10.9%)	3,206 (12.3%)	2,989 (11.5%)	< 0.001
Previous medical history (%)	15,284 (58.6%)	16,167 (62.0%)	15,862 (60.8%)	< 0.001
Statin use (%)	2,872 (11.0%)	3,530 (13.5%)	3,336 (12.8%)	< 0.001
Vitamin use (%)	5,925 (22.7%)	6,377 (24.4%)	6,086 (23.3%)	< 0.001
Inflammation				
Neutrophil count (10^9^/L)	4.0 ± 1.2	4.1 ± 1.2	4.0 ± 1.1	0.005
Monocyte count (10^9^/L)	0.5 ± 0.2	0.5 ± 0.2	0.5 ± 0.1	< 0.001
Lymphocyte count (10^9^/L)	1.9 ± 0.5	1.9 ± 0.5	1.9 ± 0.5	< 0.001
Platelet count (10^9^/L)	247 ± 51.7	251 ± 51.8	254 ± 52.0	< 0.001
SIRI	1.1 ± 0.6	1.1 ± 0.6	1.0 ± 0.6	< 0.001
SII	568 ± 255	577 ± 256	576 ± 259	< 0.001

*P* values were determined using the ANOVA test for continuous variables and the chi-square test for categorical variables. BMI, body mass index; LPA, light physical activity; MVPA, moderate-to-vigorous physical activity; SIRI, systemic inflammation response index; SII, systemic immune-inflammation index.

### 3.2 Association between rest-activity rhythm and risk of AAA

During a mean follow-up of 10.1 years, a total of 229 incident cases of AAA were recorded. Table 2 presents the associations between 13 RAR parameters and the risk of AAA. Among these parameters, four showed significant associations with AAA incidence: lower RA (HR_T1 vs. T3_ 1.49; 95% CI 1.03–2.15; Adjusted-P_trend_ = 0.023), lower M10 (HR_T1 vs. T3_ 1.51; 95% CI 1.05–2.18; Adjusted-P_trend_ = 0.013), lower amplitude (HR_T1 vs. T3_ 1.58; 95% CI 1.09–2.30; Adjusted-P_trend_ = 0.022), and lower mesor (HR_T1 vs. T3_ 1.46; 95% CI 1.04–2.14; Adjusted-P_trend_ = 0.013) were all associated with an increased risk of AAA. No significant associations were observed for the remaining parameters. The proportional hazards assumption was verified using Schoenfeld residual tests, with all *p*-values > 0.05, supporting the validity and robustness of the analyses.

**Table 2:** Association between tertiles of rest-activity rhythm and risk of AAA.

Model	Tertile 1	Tertile 2	Tertile 3	*Adjusted-*P*_trend_	Schoenfeld test (*P*-value)
IS					
Incident AAA^#^	0.307	0.261	0.298		
Model 1	1.02 (0.75–1.39)	0.94 (0.68–1.30)	Ref.	0.874	0.146
Model 2	0.91 (0.66–1.25)	0.89 (0.65–1.24)	Ref.	0.858	0.193
IV					
Incident AAA	0.215	0.307	0.344		
Model 1	Ref.	1.34 (0.96–1.88)	1.56 (1.12–2.17)	0.003	0.342
Model 2	Ref.	1.20 (0.85–1.69)	1.25 (0.89–1.76)	0.070	0.351
RA					
Incident AAA	0.473	0.241	0.157		
Model 1	1.94 (1.36–2.76)	1.23 (0.83–1.81)	Ref.	<0.001	0.605
Model 2	1.49 (1.03–2.15)	1.12 (0.75–1.65)	Ref.	0.023	0.331
L5					
Incident AAA	0.264	0.291	0.312		
Model 1	Ref.	1.03 (0.75–1.43)	1.11 (0.81–1.53)	0.349	0.331
Model 2	Ref.	0.95 (0.68–1.31)	0.91 (0.65–1.25)	0.858	0.234
M10					
Incident AAA	0.481	0.233	0.157		
Model 1	1.87 (1.31–2.66)	1.20 (0.81–1.78)	Ref.	<0.001	0.186
Model 2	1.51 (1.05–2.18)	1.09 (0.73–1.62)	Ref.	0.013	0.241
L5 start time					
Incident AAA	0.231	0.319	0.317		
Model 1	Ref.	1.38 (0.99–1.92)	1.27 (0.91–1.77)	0.602	0.743
Model 2	Ref.	1.35 (0.97–1.88)	1.18 (0.85–1.64)	0.858	0.455
M10 start time					
Incident AAA	0.321	0.293	0.252		
Model 1	1.06 (0.78–1.44)	Ref.	1.22 (0.87–1.69)	0.602	0.569
Model 2	1.08 (0.80–1.47)	Ref.	1.12 (0.81–1.57)	0.858	0.362
Pseudo-F statistic					
Incident AAA	0.268	0.307	0.292		
Model 1	1.02 (0.72–1.38)	0.94 (0.69–1.29)	Ref.	0.874	0.232
Model 2	1.03 (0.72–1.39)	0.94 (0.69–1.29)	Ref.	0.945	0.241
Amplitude					
Incident AAA	0.429	0.287	0.153		
Model 1	1.93 (1.35–2.77)	1.61 (1.10–2.36)	Ref.	<0.001	0.341
Model 2	1.58 (1.09–2.30)	1.47 (1.03–2.16)	Ref.	0.022	0.263
Mesor					
Incident AAA	0.497	0.237	0.138		
Model 1	1.82 (1.26–2.65)	1.24 (0.83–1.87)	Ref.	<0.001	0.378
Model 2	1.46 (1.04–2.14)	1.13 (0.75–1.70)	Ref.	0.013	0.311
Up mesor					
Incident AAA	0.349	0.265	0.252		
Model 1	1.04 (0.76–1.42)	Ref.	1.23 (0.88–1.71)	0.673	0.677
Model 2	1.00 (0.73–1.37)	Ref.	1.13 (0.81–1.59)	0.858	0.406
Down mesor					
Incident AAA	0.353	0.265	0.248		
Model 1	1.06 (0.78–1.45)	Ref.	1.20 (0.86–1.69)	0.936	0.679
Model 2	1.02 (0.75–1.39)	Ref.	1.11 (0.79–1.56)	0.945	0.407
Acrophase					
Incident AAA	0.349	0.264	0.252		
Model 1	1.04 (0.76–1.42)	Ref.	1.22 (0.87–1.71)	0.602	0.677
Model 2	1.00 (0.73–1.37)	Ref.	1.13 (0.81–1.59)	0.858	0.406

^#^Incidence rates per 1,000 person-years. *Adjusted *P*_trend_ refers to the *P* value after Benjamini-Hochberg correction. Model 1: adjusted for age and sex. Model 2: further adjusted for education, employment, Townsend deprivation index, shift work, BMI, medical history, smoking, diet, alcohol, and statin/vitamin use.

As shown in Supplementary Figure S2, no evidence of nonlinear dose–response relationships was observed between the four significant rest–activity rhythm parameters and AAA risk (*p* > 0.05). Subgroup analyses (Supplementary Tables S4, S5) indicated no significant interactions between the 13 rest–activity rhythm parameters and AAA incidence across subgroups defined by sex, age, or BMI. In addition, several sensitivity analyses were performed to test the robustness of the findings. As shown in Supplementary Table S6, the results remained consistent after excluding cases diagnosed within the first year of follow-up, applying competing risk models, and further adjusting for time spent in four activity categories or the PRS. These findings collectively support the consistency and robustness of our results.

### 3.3 Interactions of multiple covariates with rest-activity rhythm parameters in AAA risk

Given the potential correlations between RAR and physical activity patterns, we first examined the associations of four activity types and MVPA categories with the risk of AAA. As shown in Supplementary Tables S7, S8, after adjustment for all covariates, none of these activity measures were significantly associated with AAA incidence. In contrast, as shown in Supplementary Table S9, participants in the highest tertile of the PRS, derived using the PRS-CS method, had a significantly higher risk of AAA compared with those in the lowest tertile (HR = 3.01; 95% CI: 2.13–4.25).

**Figure 1:**
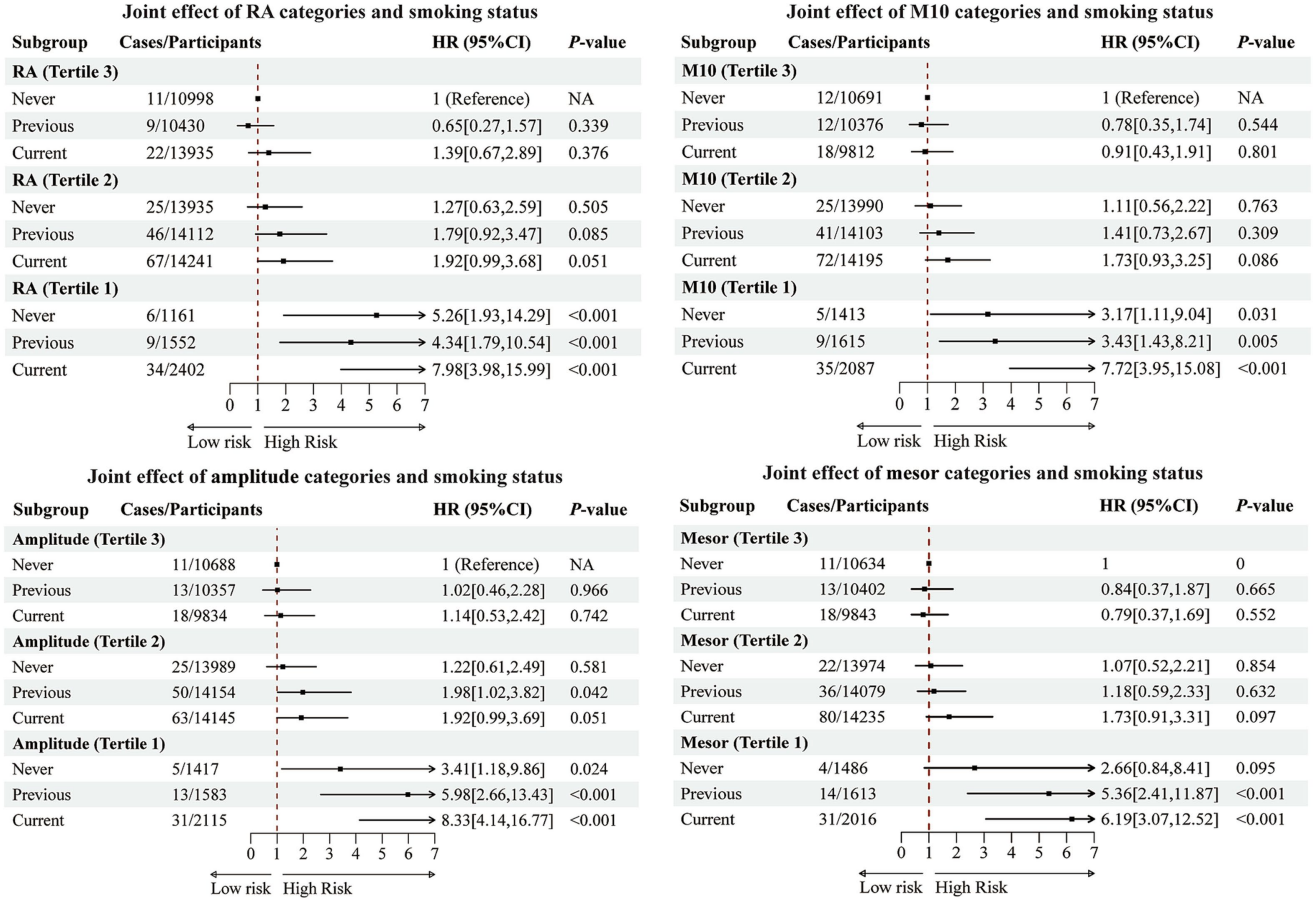
Associations of smoking and rest-activity rhythm parameters with the risk of AAA. All analyses were adjusted for age, sex, education, employment status, Townsend deprivation index, shift work, BMI, medical history, diet, alcohol consumption, and statin/vitamin use.

We further evaluated the interactions between the four rest–activity rhythm parameters that were significantly associated with AAA and several variables, including genetic risk, time spent in four activity types, MVPA categories, smoking, alcohol consumption, and dietary habits. As shown in Supplementary Tables S10, S11, no significant additive or multiplicative interactions were observed between the four rest-activity rhythm parameters and any of the variables above. However, Supplementary Table S12 shows the results of the joint effect analyses. Compared with the reference groups exhibiting the lowest AAA incidence, genetic risk, smoking, and alcohol consumption demonstrated strong joint effects with the four rest–activity rhythm parameters, whereas other activity types showed joint effects with only some of the parameters. Among these, as illustrated in Figure 1, smoking exhibited the strongest joint association. Specifically, compared with non-smokers in the highest tertile of the four rest–activity rhythm parameters, current smokers in the lowest tertile had approximately a 6- to 8-fold higher risk of developing AAA.

### 3.4 Associations of inflammatory markers and rest-activity rhythm with AAA risk

**Figure 2:**
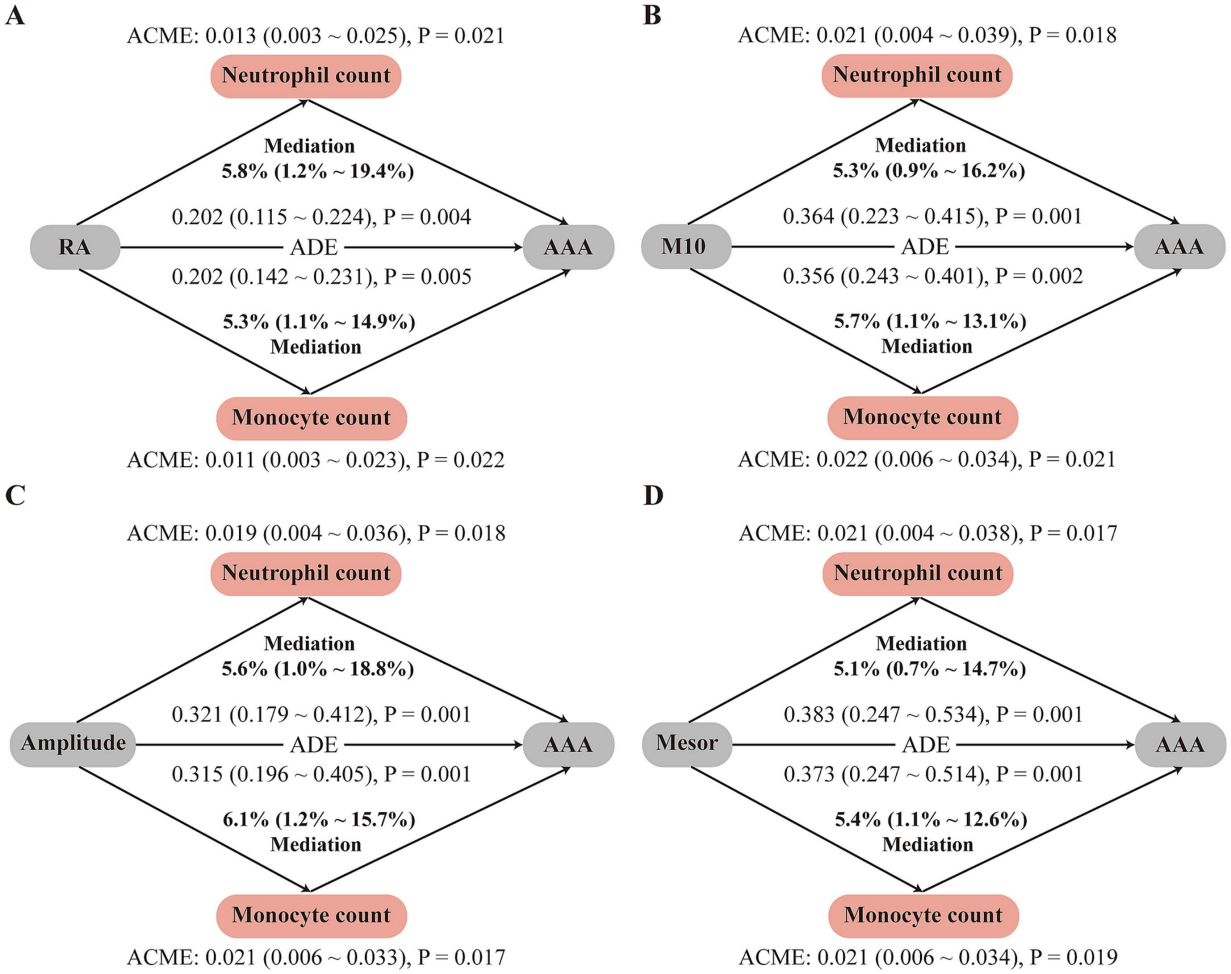
Mediating effects of inflammatory markers on the associations between rest-activity rhythm parameters and the risk of AAA. **(A)** RA; **(B)** M10; **(C)** Amplitude; **(D)** Mesor. Neutrophil count and monocyte count were evaluated as mediators in each panel. All analyses were adjusted for age, sex, education, employment status, Townsend deprivation index, shift work, BMI, medical history, smoking status, diet, alcohol consumption, and statin/vitamin use.

Given that certain rest–activity rhythm parameters may influence systemic inflammatory status and thereby affect AAA development, we further investigated the mediating effects of multiple inflammatory markers and their composite indices on the associations between the four significant RAR parameters and AAA risk. As shown in Supplementary Table S13, significant mediation effects were observed for neutrophil count, monocyte count, lymphocyte count, and the SIRI. Among these, as illustrated in Figure 2, neutrophil and monocyte counts demonstrated the strongest mediation effects, explaining approximately 5–6% of the association between rest–activity rhythm parameters and the risk of AAA (Figure 2).

### 3.5 XGBoost model performance and SHAP analysis

**Figure 3:**
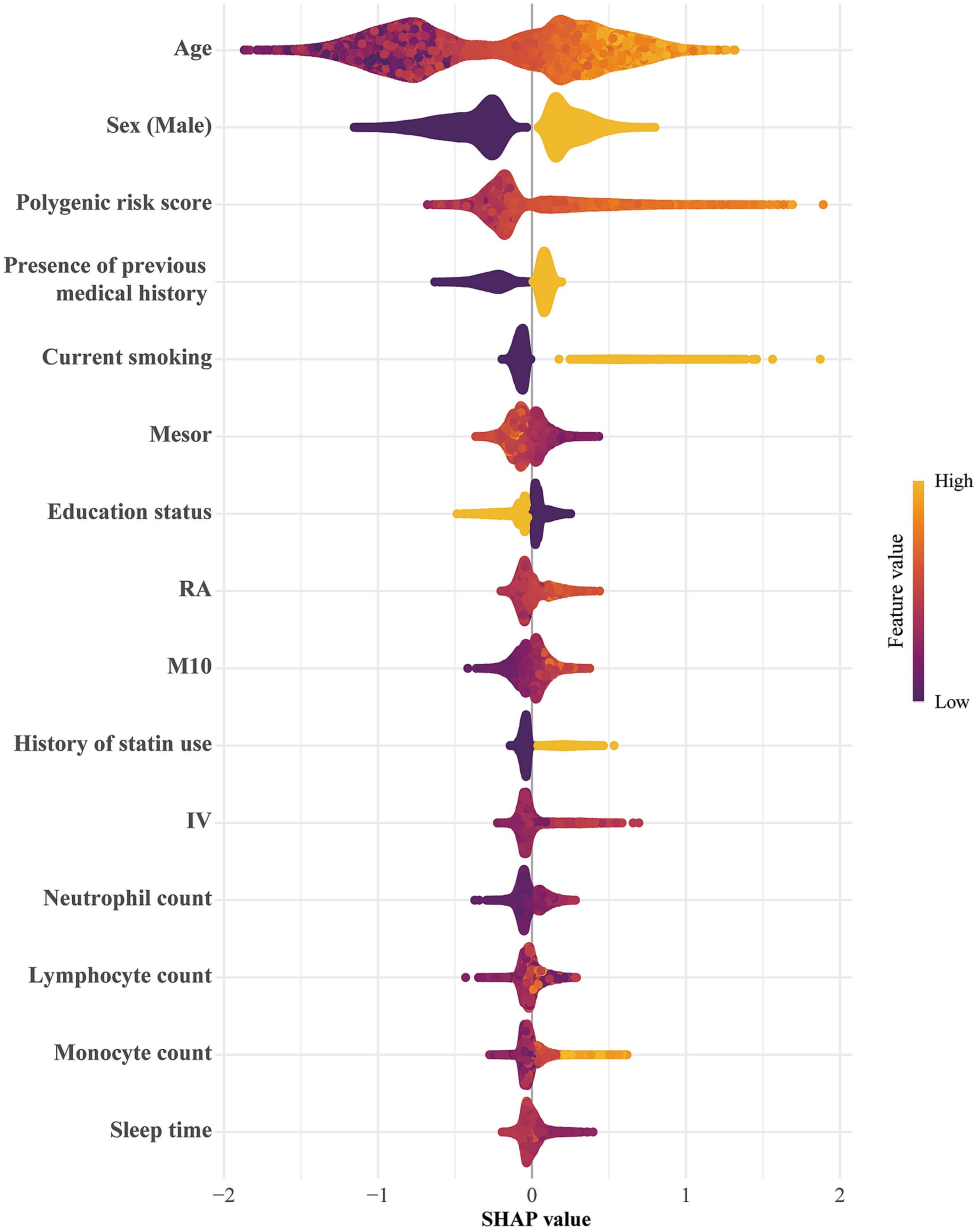
SHAP summary plot of the XGBoost model. Each point represents the SHAP value of one feature for a single participant, indicating its direction and magnitude of influence on AAA risk. The horizontal axis denotes SHAP values (positive: higher risk; negative: lower risk), and color encodes feature values (yellow: high, purple: low).

To quantify the incremental predictive contribution of RAR parameters, we compared four XGBoost models with increasing information content. In the validation dataset, the model including baseline covariates only achieved an AUC of 0.8912; adding PRS increased the AUC to 0.9214, and adding RAR increased the AUC to 0.9248. The full model incorporating both PRS and RAR showed the best discrimination, with an AUC of 0.9424. Decision curve analysis further suggested that the full model provided the greatest clinical net benefit (Supplementary Figure S3). Internal validation using five-fold cross validation showed a similar pattern (Supplementary Table S14). The full model also had the lowest Brier score (0.002709; Supplementary Figure S4), indicating the best overall predictive accuracy. SHAP based model interpretation (Supplementary Figure S5) identified the top 15 features ranked by mean absolute SHAP value, including several RAR parameters. Notably, age, sex, and genetic risk showed the greatest importance for predicting incident AAA. As shown in Figure 3, older age, male sex, and higher genetic risk were positively associated with SHAP values, indicating increased AAA risk, whereas higher Mesor, RA, and M10 were negatively associated with SHAP values, indicating lower AAA risk.

## 4 Discussion

In this study, we found that lower levels of RA, M10, amplitude, and mesor (key parameters of the RAR), were significantly associated with an increased risk of AAA. Although no significant interactions were observed between RAR parameters and accelerometer-derived activity time, MVPA categories, or genetic risk, several strong joint effects were identified, with smoking status showing the most pronounced joint association with RAR parameters. Moreover, part of the association between RAR parameters and AAA risk appeared to be mediated through low-grade systemic inflammation, with neutrophil and monocyte counts demonstrating the strongest mediating effects. Finally, within a machine learning–based survival framework integrating all covariates, the XGBoost model achieved excellent discriminative performance. The SHAP analysis revealed that age, male sex, and genetic risk were the primary contributors to AAA prediction, whereas Mesor, RA, and M10 provided meaningful incremental predictive information beyond some traditional risk factors.

RAR in our study comprised 13 parametric and nonparametric metrics. Parametric metrics characterize the 24-h pattern by fitting extended cosine models to actigraphy time series, whereas nonparametric metrics avoid model assumptions and instead summarize stability, fragmentation, and day-night contrast directly from the data; both approaches are widely used in population studies (17). In prior large cohorts, several core RAR features have been linked to adverse health outcomes, including higher risks of all-cause, cardiovascular, and cancer mortality when rhythms are weakened or fragmented (18). Evidence also extends to specific cardiovascular endpoints, for example, abnormal or blunted RAR has been associated with a higher incidence of atrial fibrillation (19). Consistent with this literature, we observed robust inverse associations between four indices closely related to “rhythmic strength/day–night contrast or daytime activation” (RA, M10, amplitude, and mesor), and subsequent risk of AAA: stronger rhythms, greater daytime activity, and higher 24-h mean activity were associated with lower AAA risk. To our knowledge, no previous prospective study has systematically reported this pattern for AAA, thus extending the spectrum of adverse outcomes linked to disrupted RAR into the AAA domain. By contrast, timing- or shape-oriented metrics (e.g., acrophase, L5/M10 onset times, upper/lower mesor, IS and IV) showed no consistent associations in our data, potentially for several reasons: the relatively small number of AAA events limited power to detect modest effects; correlations among RAR features and covariates may induce collinearity; and strength/contrast dimensions (RA, amplitude, M10, mesor) may be more proximal to relevant pathophysiology and therefore explain more variance in multivariable models, leaving less independent signal for timing/shape indices (20). Overall, our findings align with prior evidence linking lower amplitude, reduced stability, and greater fragmentation to adverse cardiovascular outcomes, while highlighting the need for larger cohorts to clarify associations for other RAR metrics with incident AAA.

Although formal tests of multiplicative or additive interaction were largely non-significant, the joint-effect analyses were clinically informative: when weak rhythmicity co-occurred with current smoking or high genetic risk, the risk of AAA increased markedly relative to the reference groups with strong rhythmicity and low exposures. This pattern is consistent with risk stacking rather than statistical interaction per se and underscores that behavioral rhythmicity does not offset conventional risk factors; instead, poor rhythms and established risks accumulate. Importantly, even where overall physical-activity categories were null, the combination of lower rhythmic strength with adverse behaviors could still yield meaningfully higher joint risk, highlighting that behavioral dose (minutes of activity) and behavioral organization (its 24-h temporal structure) represent distinct axes of vascular risk.

Our machine-learning results are complementary. The XGBoost model reaffirmed that age, sex, and a PRS play dominant roles in AAA. This is well established in human genetics and clinical epidemiology (21). Beyond these factors, the model also ranked mesor, RA, and M10 among the most informative behavioral features. SHAP values provide model-level attributions rather than causal effects (22). Nevertheless, the agreement in direction between SHAP attributions (higher mesor/RA/M10 associated with more negative SHAP values) and our Cox estimates strengthens the view that rhythmic strength and daytime activation supply additional, non-redundant information for risk stratification on top of genetics and demographics.

From a mechanistic standpoint, the observed link between weaker RAR and higher AAA risk is biologically plausible. Central and peripheral clocks coordinate diurnal oscillations in endothelial nitric oxide signaling, shear-stress responses, leukocyte trafficking, and metabolic pathways (23). Circadian disruption and dampened rhythmic amplitude impair endothelial function, increase vascular permeability, and bias immune timing toward a pro-inflammatory state (23, 24). These changes promote matrix metalloproteinase activation, extracellular-matrix degradation, and smooth muscle cell dysregulation (canonical elements of aneurysm pathogenesis) (25). In our mediation analysis, neutrophil and monocyte counts were implicated, consistent with clock-regulated low-grade inflammation partially transmitting the effect of disrupted behavioral rhythms to aneurysmal remodeling. Taken together, circadian pathways complement the current multifactorial model of AAA biology rather than replace it.

This study has several notable strengths. First, it was based on a large-scale prospective cohort design, which enhances the robustness and reliability of the findings. Second, both parametric and nonparametric approaches were systematically applied to comprehensively evaluate the relative importance of different RAR parameters in predicting AAA risk. Furthermore, to our knowledge, this is the first prospective study to investigate the potential mediating roles of RAR parameters, physical activity patterns, and inflammatory markers in the development of AAA. These findings provide new insights and valuable guidance for future preventive strategies.

However, several limitations should be acknowledged. First, the UK Biobank lacks detailed information on aneurysm diameter, which prevented us from examining aneurysm size as an outcome. Second, although we adjusted for a wide range of potential confounders, residual confounding (e.g., from unmeasured comorbidities) cannot be fully ruled out. Third, the study population was restricted to White British participants who completed dietary assessments, which may limit the generalizability of our findings to other racial or ethnic groups. Future large-scale prospective studies in more diverse populations are warranted to replicate these analyses. Fourth, because inflammatory biomarkers were measured before accelerometer assessment, the mediation analysis may be constrained by the temporal ordering of measurements. Therefore, further validation in larger prospective cohort studies with a more clearly defined measurement timeline is needed to clarify these relationships. Finally, accelerometer recordings were obtained only once over a 5-7 day period, and thus the observed rest-activity rhythms may not fully capture longitudinal changes in behavioral patterns. Given the importance of RAR for population health, future studies should incorporate repeated assessments of key RAR parameters to better understand their dynamic relationships with AAA risk and other health outcomes.

## 5 Conclusion

In summary, our findings indicate that weaker rest-activity rhythms (particularly lower RA, mesor, M10, and amplitude) are closely associated with a higher risk of abdominal aortic aneurysm. Part of this association appears to be mediated by chronic inflammatory markers, chiefly neutrophil and monocyte counts. Moreover, the combination of weaker rhythmicity with current smoking or high genetic risk substantially amplifies AAA risk. These results underscore that integrating objective rest–activity rhythm metrics with established risk factors can enhance risk stratification for AAA and inform targeted preventive strategies.

## Data Availability

Publicly available datasets were analyzed in this study. This data can be found at: this study was conducted under UK Biobank application number 145937. The UK Biobank data are available on application to the UK Biobank (www.ukbiobank.ac.uk/).

## Glossary

AAA: Abdominal aortic aneurysm
RAR: Rest–activity rhythm
RA: Relative amplitude
M10: Most active 10-h period
L5: Least active 5-h period
MESOR: Midline estimating statistic of rhythm
IS: Interdaily stability
IV: Intradaily variability
PRS: Polygenic risk score
PRS-CS: Polygenic risk score–continuous shrinkage
LPA: Light physical activity
MVPA: Moderate-to-vigorous physical activity
BMI: Body mass index
RCS: Restricted cubic spline
SHAP: SHapley Additive exPlanations
XGBoost: Extreme Gradient Boosting
UKB: UK Biobank
GWAS: Genome-wide association study
LD: Linkage disequilibrium
SNP: Single-nucleotide polymorphism
ICD-10: International Classification of Diseases, 10th Revision
C-index: Concordance index
CI: Confidence interval
HR: Hazard ratio
SIRI: Systemic inflammation response index
SII: Systemic immune–inflammation index
WHO: World Health Organization
TDI: Townsend Deprivation Index
SD: Standard deviation
RERI: Relative excess risk due to interaction
