# Supplementary material for: Accelerometer-derived rest-activity rhythms, genetic risk, and chronic inflammatory markers and the risk of abdominal aortic aneurysm
Source: Front Public Health. 2026 Apr 30;14:1800800. doi: 10.3389/fpubh.2026.1800800 (PMC13171580; doi:10.3389/fpubh.2026.1800800)
Supplement: Supplementary file 1 [file Supplementary_file_1.DOCX]

**Supplementary Material**

**Supplementary method**

**Supplementary Table S1.** Description of parametric and nonparametric rest-activity rhythm parameters

**Supplementary Table S2.** Components of an ideal diet

**Supplementary Table S3.** Collinearity diagnostics for covariates included in Cox model 2

**Supplementary Table S4.** Subgroup associations between rest-activity rhythm and AAA

**Supplementary Table S5.** Interactions between rest-activity rhythm and subgroups

**Supplementary Table S6.** Sensitivity analyses of the association between rest-activity rhythm and risk of AAA

**Supplementary Table S7.** Association between daily activity time categories and risk of AAA

**Supplementary Table S8.** Association between MVPA categories and risk of AAA

**Supplementary Table S9.** Association between PRS and risk of AAA

**Supplementary Table S10.** Multiplicative interactions between rest-activity rhythm and multiple variables

**Supplementary Table S11.** Additive interactions between rest-activity rhythm and multiple variables

**Supplementary Table S12.** Joint interactions between rest–activity rhythm and multiple variables

**Supplementary Table S13.** Mediating effects of inflammatory markers on the association between rest-activity rhythm parameters and the risk of AAA

**Supplementary Table S14.** Predictive performance of four XGBoost models for abdominal aortic aneurysm

**Supplementary Figure S1.** Flowchart of study participants

**Supplementary Figure S2.** Restricted cubic spline analyses of the associations between four rest–activity rhythm parameters and the risk of AAA

**Supplementary Figure S3.** ROC and decision curve analyses of four XGBoost models for AAA prediction

**Supplementary Figure S4.** Calibration plots of four XGBoost models for AAA prediction

**Supplementary Figure S5.** Feature importance ranked by mean absolute SHAP values in the XGBoost model

**Supplementary Table S1.** Description of parametric and nonparametric rest-activity rhythm parameters

| **Category** | **Parameter** | **Description** |
| --- | --- | --- |
| **Nonparametric parameters** | Interdaily stability (IS) | Quantifies the consistency of daily activity patterns by comparing hour-to-hour variations across days. Greater IS values indicate more stable and regular day-to-day rhythms. |
|  | Intradaily variability (IV) | Measures how fragmented the daily rhythm is by evaluating fluctuations around the hourly mean. Higher IV reflects greater irregularity, such as frequent naps or nighttime awakenings. |
|  | Relative amplitude (RA) | Calculated as (M10 − L5)/(M10 + L5), representing the contrast between daytime and nighttime activity. Higher RA denotes stronger day–night differentiation and healthier rhythm strength. |
|  | L5 | The mean activity level during the five consecutive hours of minimum movement within a day, typically reflecting nighttime rest intensity. |
|  | M10 | The mean activity level during the ten consecutive hours with the highest activity, generally indicating daytime activity levels. |
|  | L5 start time | The onset time of the least active five-hour period, marking the beginning of the daily rest phase. |
|  | M10 start time | The onset time of the most active ten-hour period, marking the start of the primary daytime activity phase. |
| **Parametric parameters** | Pseudo-F-statistic | A statistical indicator reflecting the overall robustness of circadian rhythmicity. Higher F values indicate that the activity pattern fits the rhythm model more strongly, suggesting greater rhythm regularity. |
|  | Amplitude | The gap between the highest and lowest fitted activity levels, representing rhythm strength. Larger amplitudes correspond to stronger day–night contrasts in activity. |
|  | Mesor | The midline of the fitted rhythm curve, representing the average activity intensity across the 24-hour cycle. |
|  | Up mesor | The time point when activity shifts from a low to high level; smaller values denote an earlier rise in activity, indicating a more advanced rhythm phase. |
|  | Down mesor | The time point when activity declines from high to low levels; smaller values suggest an earlier reduction in activity, reflecting an earlier circadian timing. |
|  | Acrophase | The timing of peak activity within the fitted curve. Higher values indicate a later daily peak. |

**Supplementary Table S2.** Components of an ideal diet

| **Diet component** | **Field IDs** | **Intake goal** |
| --- | --- | --- |
| Fruit | 1309 1319 | ≥3 servings/day |
| Vegetable | 1289 1299 | ≥3 servings/day |
| Whole grains | 1438/1448 1458/1468 | ≥3 servings/day |
| Fish (Shell) | 1329 1339 | ≥2 servings/day |
| Dairy | 1408 1418 | ≥2 servings/day |
| Vegetable oils | 1428 1438 2654 | ≥2 servings/day |
| Refined grains | 1438/1448 1458/1468 | ≤2 servings/day |
| processed meats | 1349 3680 | ≤1 servings/day |
| Unprocessed meats | 1359 1369 1379 1389 3680 | ≤2 servings/day |
| Sugar-sweetened beverages | 6144 | No consumption |

**Supplementary Table S3.** Collinearity diagnostics for covariates included in Cox model 2

| **Variable** | **GVIF** |
| --- | --- |
| Sex | 1.042 |
| Age | 1.145 |
| Education | 1.036 |
| Employment status | 1.057 |
| Townsend deprivation index | 1.043 |
| BMI | 1.093 |
| Shift work | 1.066 |
| Medical history | 1.088 |
| Statin use | 1.116 |
| Vitmin use | 1.017 |
| Smoking status | 1.081 |
| Diet | 1.009 |
| Alcohol consumption | 1.027 |

Multicollinearity among covariates included in Cox model 2 was assessed using generalized variance inflation factors (GVIFs).

**Supplementary Table S4.** Subgroup associations between rest-activity rhythm and AAA

| **Subgroup** | **Tertile 1** | **Tertile 2** | **Tertile 3** | ***Adjusted-*P*_trend_** | **Schoenfeld Test (P-value)** |
| --- | --- | --- | --- | --- | --- |
| **Sex (Male)** |  |  |  |  |  |
| IS | 0.88 (0.62-1.23) | 0.82 (0.57-1.18) | Ref. | 0.803 | 0.107 |
| IV | Ref. | 1.25 (0.86-1.83) | 1.26 (0.87-1.83) | 0.125 | 0.212 |
| RA | 1.32 (0.88-1.97) | 1.09 (0.71-1.67) | Ref. | 0.107 | 0.281 |
| L5 | Ref. | 0.88 (0.62-1.25) | 0.82 (0.58-1.17) | 0.973 | 0.207 |
| M10 | 1.45 (1.02-2.18) | 1.09 (0.71-1.68) | Ref. | 0.045 | 0.169 |
| L5 start time | Ref. | 1.28 (0.89-1.83) | 1.18 (0.83-1.69) | 0.803 | 0.281 |
| M10 start time | 1.00 (0.71-1.39) | Ref. | 1.07 (0.75-1.54) | 0.803 | 0.276 |
| Pseudo-F statistic | 0.99 (0.70-1.41) | 0.86 (0.60-1.21) | Ref. | 0.888 | 0.171 |
| Amplitude | 1.48 (1.02-2.21) | 1.38 (0.90-2.09) | Ref. | 0.045 | 0.168 |
| Mesor | 1.54 (1.04-2.39) | 1.27 (0.80-2.03) | Ref. | 0.026 | 0.172 |
| Up mesor | 1.06 (0.75-1.50) | Ref. | 1.23 (0.84-1.79) | 0.803 | 0.313 |
| Down mesor | 1.09 (0.77-1.53) | Ref. | 1.20 (0.82-1.75) | 0.973 | 0.213 |
| Acrophase | 1.06 (0.75-1.50) | Ref. | 1.22 (0.84-1.78) | 0.803 | 0.316 |
| **Sex (Female)** |  |  |  |  |  |
| IS | 1.05 (0.44-2.51) | 1.29 (0.6-2.79) | Ref. | 0.772 | 0.493 |
| IV | Ref. | 1.03 (0.47-2.26) | 1.41 (0.62-3.18) | 0.372 | 0.582 |
| RA | 2.50 (1.01-6.18) | 1.20 (0.45-3.19) | Ref. | 0.113 | 0.473 |
| L5 | Ref. | 1.49 (0.63-3.50) | 1.79 (0.77-4.13) | 0.394 | 0.537 |
| M10 | 1.71 (0.71-4.12) | 1.11 (0.44-2.79) | Ref. | 0.263 | 0.566 |
| L5 start time | Ref. | 1.81 (0.78-4.21) | 1.23 (0.51-3.03) | 0.897 | 0.277 |
| M10 start time | 1.67 (0.76-3.69) | Ref. | 1.39 (0.61-3.19) | 0.782 | 0.568 |
| Pseudo-F statistic | 0.84 (0.32-2.22) | 1.36 (0.67-2.75) | Ref. | 0.831 | 0.559 |
| Amplitude | 2.14 (0.81-5.68) | 2.03 (0.78-5.27) | Ref. | 0.211 | 0.502 |
| Mesor | 1.19 (0.52-2.73) | 0.70 (0.28-1.74) | Ref. | 0.565 | 0.574 |
| Up mesor | 0.72 (0.32-1.58) | Ref. | 0.78 (0.36-1.69) | 0.982 | 0.556 |
| Down mesor | 0.72 (0.33-1.59) | Ref. | 0.78 (0.36-1.69) | 0.781 | 0.567 |
| Acrophase | 0.72 (0.33-1.59) | Ref. | 0.78 (0.36-1.69) | 0.983 | 0.541 |
| **Age (60)** |  |  |  |  |  |
| IS | 0.96 (0.67-1.36) | 0.97 (0.68-1.39) | Ref. | 0.987 | 0.373 |
| IV | Ref. | 1.25 (0.86-1.82) | 1.27 (0.87-1.85) | 0.159 | 0.433 |
| RA | 1.51 (1.03-2.31) | 1.13 (0.73-1.77) | Ref. | 0.026 | 0.471 |
| L5 | Ref. | 0.94 (0.66-1.35) | 0.91 (0.64-1.31) | 0.801 | 0.425 |
| M10 | 1.52 (1.01-2.30) | 1.01 (0.64-1.60) | Ref. | 0.013 | 0.384 |
| L5 start time | Ref. | 1.53 (1.05-2.22) | 1.31 (0.90-1.91) | 0.691 | 0.442 |
| M10 start time | 1.01 (0.72-1.42) | Ref. | 1.13 (0.78-1.63) | 0.711 | 0.444 |
| Pseudo-F statistic | 1.10 (0.77-1.59) | 0.91 (0.64-1.30) | Ref. | 0.801 | 0.411 |
| Amplitude | 1.64 (1.07-2.52) | 1.49 (0.96-2.32) | Ref. | 0.013 | 0.401 |
| Mesor | 1.67 (1.05-2.65) | 1.26 (0.77-2.07) | Ref. | 0.001 | 0.393 |
| Up mesor | 0.94 (0.67-1.32) | Ref. | 1.04 (0.71-1.52) | 0.750 | 0.438 |
| Down mesor | 0.95 (0.68-1.33) | Ref. | 1.03 (0.70-1.50) | 0.691 | 0.437 |
| Acrophase | 0.94 (0.67-1.32) | Ref. | 1.03 (0.71-1.51) | 0.471 | 0.436 |
| **Age (59)** |  |  |  |  |  |
| IS | 0.78 (0.39-1.58) | 0.66 (0.31-1.41) | Ref. | 0.869 | 0.479 |
| IV | Ref. | 1.05 (0.46-2.39) | 1.45 (0.68-3.06) | 0.869 | 0.427 |
| RA | 1.51 (0.70-3.26) | 1.09 (0.48-2.47) | Ref. | 0.331 | 0.504 |
| L5 | Ref. | 1.02 (0.47-2.21) | 1.03 (0.48-2.21) | 0.772 | 0.485 |
| M10 | 1.48 (0.67-3.24) | 1.43 (0.65-3.12) | Ref. | 0.409 | 0.488 |
| L5 start time | Ref. | 0.82 (0.39-1.70) | 0.84 (0.41-1.72) | 0.752 | 0.467 |
| M10 start time | 1.56 (0.73-3.35) | Ref. | 1.25 (0.57-2.74) | 0.807 | 0.423 |
| Pseudo-F statistic | 0.69 (0.31-1.54) | 1.04 (0.52-2.10) | Ref. | 0.409 | 0.488 |
| Amplitude | 1.51 (0.69-3.30) | 1.47 (0.67-3.21) | Ref. | 0.535 | 0.489 |
| Mesor | 1.05 (0.49-2.21) | 0.91 (0.43-1.95) | Ref. | 0.454 | 0.466 |
| Up mesor | 1.40 (0.63-3.14) | Ref. | 1.66 (0.76-3.62) | 0.992 | 0.441 |
| Down mesor | 1.49 (0.67-3.31) | Ref. | 1.59 (0.72-3.49) | 0.174 | 0.491 |
| Acrophase | 1.40 (0.63-3.14) | Ref. | 1.67 (0.77-3.63) | 0.922 | 0.442 |
| **BMI (Normal)** |  |  |  |  |  |
| IS | 1.01 (0.42-2.42) | 1.11 (0.48-2.57) | Ref. | 0.631 | 0.613 |
| IV | Ref. | 0.94 (0.39-2.27) | 1.29 (0.56-2.96) | 0.731 | 0.647 |
| RA | 2.46 (1.04-6.19) | 1.30 (0.48-3.51) | Ref. | 0.061 | 0.612 |
| L5 | Ref. | 1.33 (0.56-3.17) | 1.37 (0.56-3.33) | 0.484 | 0.646 |
| M10 | 1.28 (0.54-3.03) | 0.87 (0.35-2.16) | Ref. | 0.093 | 0.634 |
| L5 start time | Ref. | 1.17 (0.48-2.82) | 1.14 (0.48-2.71) | 0.693 | 0.456 |
| M10 start time | 0.66 (0.27-1.59) | Ref. | 1.21 (0.54-2.72) | 0.208 | 0.629 |
| Pseudo-F statistic | 1.05 (0.42-2.61) | 1.13 (0.50-2.59) | Ref. | 0.966 | 0.575 |
| Amplitude | 1.97 (0.71-5.46) | 2.56 (0.98-6.65) | Ref. | 0.079 | 0.618 |
| Mesor | 1.83 (0.71-4.69) | 1.31 (0.49-3.48) | Ref. | 0.118 | 0.635 |
| Up mesor | 1.02 (0.42-2.46) | Ref. | 1.57 (0.67-3.68) | 0.508 | 0.568 |
| Down mesor | 1.03 (0.42-2.48) | Ref. | 1.58 (0.68-3.69) | 0.328 | 0.522 |
| Acrophase | 1.02 (0.42-2.45) | Ref. | 1.57 (0.67-3.67) | 0.416 | 0.541 |
| **BMI (Abnormal)** | |  |  |  |  |
| IS | 0.89 (0.64-1.25) | 0.86 (0.61-1.23) | Ref. | 0.905 | 0.169 |
| IV | Ref. | 1.26 (0.87-1.83) | 1.30 (0.90-1.88) | 0.078 | 0.174 |
| RA | 1.35 (1.01-2.02) | 1.06 (0.70-1.63) | Ref. | 0.045 | 0.238 |
| L5 | Ref. | 0.90 (0.63-1.28) | 0.88 (0.62-1.24) | 0.875 | 0.201 |
| M10 | 1.57 (1.04-2.37) | 1.15 (0.74-1.79) | Ref. | 0.047 | 0.158 |
| L5 start time | Ref. | 1.39 (0.98-1.98) | 1.20 (0.84-1.72) | 0.543 | 0.218 |
| M10 start time | 1.16 (0.83-1.61) | Ref. | 1.11 (0.77-1.60) | 0.905 | 0.187 |
| Pseudo-F statistic | 1.01 (0.70-1.42) | 0.91 (0.65-1.28) | Ref. | 0.858 | 0.184 |
| Amplitude | 1.50 (1.01-2.24) | 1.31 (0.86-1.99) | Ref. | 0.023 | 0.164 |
| Mesor | 1.38 (1.02-2.11) | 1.08 (0.69-1.69) | Ref. | 0.042 | 0.149 |
| Up mesor | 0.99 (0.71-1.39) | Ref. | 1.07 (0.74-1.55) | 0.844 | 0.209 |
| Down mesor | 1.01 (0.73-1.41) | Ref. | 1.05 (0.73-1.52) | 0.783 | 0.214 |
| Acrophase | 0.99 (0.71-1.39) | Ref. | 1.07 (0.74-1.55) | 0.746 | 0.209 |

*Adjusted P_trend_ refers to the P value after Benjamini-Hochberg correction. All analyses were adjusted for age, sex, education, employment status, Townsend deprivation index, shift work, BMI, medical history, smoking status, diet, alcohol consumption, and statin/vitamin use, except for the stratified variable in each subgroup analysis.

**Supplementary Table S5.** Interactions between rest-activity rhythm and subgroups

| **Variable** | **Sex (P_interaction_)** | **Age (P_interaction_)** | **BMI (P_interaction_)** |
| --- | --- | --- | --- |
| IS | 0.648 | 0.331 | 0.732 |
| IV | 0.743 | 0.621 | 0.546 |
| RA | 0.684 | 0.339 | 0.343 |
| L5 | 0.394 | 0.623 | 0.567 |
| M10 | 0.637 | 0.316 | 0.531 |
| L5 start time | 0.909 | 0.602 | 0.884 |
| M10 start time | 0.951 | 0.354 | 0.187 |
| Pseudo-F statistic | 0.519 | 0.958 | 0.914 |
| Amplitude | 0.909 | 0.153 | 0.392 |
| Mesor | 0.178 | 0.409 | 0.716 |
| Up mesor | 0.647 | 0.516 | 0.441 |
| Down mesor | 0.438 | 0.239 | 0.233 |
| Acrophase | 0.661 | 0.559 | 0.395 |

**Supplementary Table S6.** Sensitivity analyses of the association between rest-activity rhythm and risk of AAA

| **Analyses** | **Tertile 1** | **Tertile 2** | **Tertile 3** | ***Adjusted-*P*_trend_** | **Schoenfeld Test (P-value)** |
| --- | --- | --- | --- | --- | --- |
| **Excluding events that occurred within the first years of follow-up** | | | | | |
| IS | 0.87 (0.63-1.22) | 0.87 (0.62-1.22) | Ref. | 0.823 | 0.184 |
| IV | Ref. | 1.27 (0.89-1.81) | 1.30 (0.91-1.86) | 0.107 | 0.127 |
| RA | 1.46 (1.01-2.15) | 1.08 (0.71-1.62) | Ref. | 0.041 | 0.199 |
| L5 | Ref. | 1.05 (0.74-1.49) | 1.10 (0.78-1.55) | 0.823 | 0.194 |
| M10 | 1.46 (1.02-2.15) | 1.17 (0.77-1.76) | Ref. | 0.106 | 0.194 |
| L5 start time | Ref. | 1.43 (1.01-2.02) | 1.26 (0.89-1.79) | 0.823 | 0.192 |
| M10 start time | 1.06 (0.77-1.46) | Ref. | 1.07 (0.76-1.52) | 0.895 | 0.174 |
| Pseudo-F statistic | 0.91 (0.64-1.29) | 0.99 (0.72-1.38) | Ref. | 0.823 | 0.193 |
| Amplitude | 1.52 (1.03-2.24) | 1.47 (0.99-2.19) | Ref. | 0.107 | 0.194 |
| Mesor | 1.38 (1.01-2.06) | 1.18 (0.77-1.81) | Ref. | 0.023 | 0.195 |
| Up mesor | 0.97 (0.70-1.35) | Ref. | 1.13 (0.79-1.61) | 0.823 | 0.193 |
| Down mesor | 0.99 (0.72-1.38) | Ref. | 1.11 (0.78-1.58) | 0.988 | 0.191 |
| Acrophase | 0.97 (0.70-1.35) | Ref. | 1.13 (0.79-1.60) | 0.823 | 0.193 |
| **Competing risk regression model** | | | | | |
| IS | 0.91 (0.67-1.24) | 0.90 (0.65-1.25) | Ref. | 0.817 | NA |
| IV | Ref. | 1.22 (0.87-1.71) | 1.31 (0.94-1.82) | 0.044 | NA |
| RA | 1.49 (1.03-2.16) | 1.12 (0.76-1.66) | Ref. | 0.017 | NA |
| L5 | Ref. | 0.96 (0.69-1.33) | 0.94 (0.67-1.31) | 0.817 | NA |
| M10 | 1.51 (1.05-2.16) | 1.09 (0.74-1.63) | Ref. | 0.017 | NA |
| L5 start time | Ref. | 1.36 (0.98-1.89) | 1.20 (0.87-1.67) | 0.817 | NA |
| M10 start time | 1.06 (0.78-1.45) | Ref. | 1.13 (0.81-1.58) | 0.817 | NA |
| Pseudo-F statistic | 0.99 (0.72-1.38) | 0.94 (0.68-1.29) | Ref. | 0.889 | NA |
| Amplitude | 1.58 (1.09-2.28) | 1.48 (1.01-2.16) | Ref. | 0.023 | NA |
| Mesor | 1.47 (1.01-2.14) | 1.14 (0.76-1.71) | Ref. | 0.013 | NA |
| Up mesor | 0.99 (0.73-1.36) | Ref. | 1.16 (0.82-1.62) | 0.817 | NA |
| Down mesor | 1.02 (0.75-1.39) | Ref. | 1.14 (0.81-1.59) | 0.891 | NA |
| Acrophase | 0.99 (0.73-1.36) | Ref. | 1.15 (0.82-1.62) | 0.816 | NA |
| **Additionally adjusted for PRS** | | | | | |
| IS | 0.90 (0.66-1.23) | 0.88 (0.64-1.22) | Ref. | 0.807 | 0.333 |
| IV | Ref. | 1.22 (0.87-1.72) | 1.31 (0.93-1.83) | 0.072 | 0.329 |
| RA | 1.49 (1.03-2.15) | 1.12 (0.75-1.65) | Ref. | 0.019 | 0.371 |
| L5 | Ref. | 0.94 (0.68-1.30) | 0.94 (0.68-1.29) | 0.807 | 0.312 |
| M10 | 1.52 (1.05-2.19) | 1.09 (0.73-1.61) | Ref. | 0.013 | 0.337 |
| L5 start time | Ref. | 1.39 (1.01-1.93) | 1.21 (0.87-1.69) | 0.807 | 0.338 |
| M10 start time | 1.08 (0.80-1.47) | Ref. | 1.15 (0.82-1.60) | 0.807 | 0.324 |
| Pseudo-F statistic | 1.01 (0.72-1.40) | 0.98 (0.71-1.34) | Ref. | 0.906 | 0.341 |
| Amplitude | 1.59 (1.09-2.30) | 1.47 (1.01-2.15) | Ref. | 0.017 | 0.344 |
| Mesor | 1.45 (1.03-2.12) | 1.11 (0.74-1.68) | Ref. | 0.013 | 0.327 |
| Up mesor | 1.01 (0.73-1.37) | Ref. | 1.15 (0.82-1.61) | 0.807 | 0.344 |
| Down mesor | 1.02 (0.75-1.40) | Ref. | 1.14 (0.81-1.60) | 0.906 | 0.343 |
| Acrophase | 1.02 (0.73-1.37) | Ref. | 1.15 (0.82-1.61) | 0.807 | 0.341 |
| **Additionally adjusted for LPA, MVPA, sedentary time, and sleep** | | | | | |
| IS | 0.71 (0.50-1.03) | 0.79 (0.56-1.11) | Ref. | 0.302 | 0.293 |
| IV | Ref. | 1.18 (0.83-1.68) | 1.22 (0.84-1.77) | 0.211 | 0.289 |
| RA | 1.34 (0.86-2.08) | 1.06 (0.71-1.59) | Ref. | 0.208 | 0.315 |
| L5 | Ref. | 0.96 (0.70-1.33) | 0.95 (0.68-1.32) | 0.889 | 0.258 |
| M10 | 1.43 (1.01-2.39) | 1.06 (0.68-1.63) | Ref. | 0.037 | 0.275 |
| L5 start time | Ref. | 1.34 (0.97-1.86) | 1.17 (0.84-1.63) | 0.840 | 0.291 |
| M10 start time | 1.10 (0.81-1.50) | Ref. | 1.10 (0.79-1.54) | 0.951 | 0.277 |
| Pseudo-F statistic | 0.82 (0.56-1.20) | 0.84 (0.60-1.17) | Ref. | 0.303 | 0.298 |
| Amplitude | 1.55 (1.05-2.52) | 1.40 (0.92-2.12) | Ref. | 0.041 | 0.291 |
| Mesor | 1.48 (1.02-2.43) | 1.06 (0.68-1.67) | Ref. | 0.026 | 0.244 |
| Up mesor | 1.01 (0.74-1.39) | Ref. | 1.12 (0.80-1.57) | 0.951 | 0.291 |
| Down mesor | 1.03 (0.75-1.41) | Ref. | 1.11 (0.78-1.54) | 0.951 | 0.291 |
| Acrophase | 1.01 (0.74-1.39) | Ref. | 1.12 (0.81-1.56) | 0.918 | 0.291 |
| **Multiple imputation** | | | | | |
| IS | 0.90 (0.66-1.24) | 0.89 (0.65-1.23) | Ref. | 0.924 | 0.254 |
| IV | Ref. | 1.20 (0.85-1.68) | 1.30 (0.93-1.81) | 0.081 | 0.224 |
| RA | 1.53 (1.06-2.21) | 1.12 (0.76-1.66) | Ref. | 0.013 | 0.301 |
| L5 | Ref. | 0.97 (0.70-1.34) | 0.95 (0.69-1.31) | 0.924 | 0.251 |
| M10 | 1.53 (1.06-2.20) | 1.13 (0.76-1.68) | Ref. | 0.013 | 0.206 |
| L5 start time | Ref. | 1.38 (0.99-1.91) | 1.21 (0.87-1.69) | 0.924 | 0.263 |
| M10 start time | 1.09 (0.80-1.49) | Ref. | 1.14 (0.82-1.58) | 0.924 | 0.267 |
| Pseudo-F statistic | 1.01 (0.72-1.40) | 0.93 (0.68-1.28) | Ref. | 0.944 | 0.264 |
| Amplitude | 1.52 (1.06-2.19) | 1.41 (0.97-2.05) | Ref. | 0.013 | 0.252 |
| Mesor | 1.40 (1.01-2.04) | 1.08 (0.72-1.61) | Ref. | 0.013 | 0.205 |
| Up mesor | 1.01 (0.73-1.36) | Ref. | 1.13 (0.81-1.58) | 0.924 | 0.268 |
| Down mesor | 1.05 (0.77-1.43) | Ref. | 1.11 (0.79-1.56) | 0.999 | 0.273 |
| Acrophase | 0.98 (0.72-1.34) | Ref. | 1.10 (0.79-1.54) | 0.924 | 0.268 |

*Adjusted P_trend_ refers to the P value after Benjamini-Hochberg correction. All analyses were adjusted for age, sex, education, employment status, Townsend deprivation index, shift work, BMI, medical history, smoking status, diet, alcohol consumption, and statin/vitamin use.

**Supplementary Table S7.** Association between daily activity time categories and risk of AAA

| **Model** | **Tertile 1** | **Tertile 2** | **Tertile 3** | ***P*_trend_** | **Schoenfeld Test (P-value)** |
| --- | --- | --- | --- | --- | --- |
| **LPA time** |  |  |  |  |  |
| Incident AAA^#^ | 0.411 | 0.262 | 0.196 |  |  |
| Model 1 | 1.25 (0.89-1.75) | 1.01 (0.71-1.45) | Ref. | 0.038 | 0.729 |
| Model 2 | 1.15 (0.82-1.62) | 1.01 (0.69-1.43) | Ref. | 0.164 | 0.416 |
| **MVPA time** |  |  |  |  |  |
| Incident AAA | 0.358 | 0.244 | 0.267 |  |  |
| Model 1 | 1.57 (1.14-2.14) | 1.02 (0.73-1.42) | Ref. | 0.001 | 0.065 |
| Model 2 | 1.15 (0.83-1.59) | 0.92 (0.65-1.29) | Ref. | 0.134 | 0.138 |
| **Sedentary time** |  |  |  |  |  |
| Incident AAA | 0.199 | 0.301 | 0.369 |  |  |
| Model 1 | Ref. | 1.26 (0.89-1.78) | 1.35 (0.97-1.90) | 0.021 | 0.309 |
| Model 2 | Ref. | 1.24 (0.87-1.76) | 1.22 (0.87-1.73) | 0.143 | 0.315 |
| **Sleep time** |  |  |  |  |  |
| Incident AAA | 0.304 | 0.228 | 0.335 |  |  |
| Model 1 | 1.30 (0.93-1.81) | Ref. | 1.29 (0.93-1.79) | 0.478 | 0.471 |
| Model 2 | 1.29 (0.93-1.81) | Ref. | 1.22 (0.88-1.70) | 0.792 | 0.313 |

^#^Incidence rates per 1,000 person-years. Model 1: adjusted for age and sex. Model 2: further adjusted for education, employment, Townsend deprivation index, shift work, BMI, medical history, smoking, diet, alcohol, and statin/vitamin use. Abbreviations: LPA, Light Physical Activity; MVPA, Moderate-to-Vigorous Physical Activity.

**Supplementary Table S8.** Association between MVPA categories and risk of AAA

| **Group** | **Incident AAA^#^** | **Model 1** | **Model 2** |
| --- | --- | --- | --- |
| **MVPA (WHO activity level classification)** | | |  |
| Inactive (<150 min/week) | 0.355 | 1.53 (1.18-2.01) | 1.19 (0.90-1.57) |
| Active (≥150 min/week) | 0.255 | Ref. | Ref. |
| P_trend_ |  | 0.001 | 0.221 |
| Schoenfeld Test (P-value) |  | 0.103 | 0.129 |
| **MVPA (Weekly activity pattern)** | |  |  |
| Inactive | 0.355 | 1.57 (1.09-2.26) | 1.21 (0.83-1.76) |
| Regularly active | 0.269 | Ref. | Ref. |
| Weekend warrior | 0.232 | 1.03 (0.72-1.48) | 1.02 (0.71-1.47) |
| P_trend_ |  | 0.006 | 0.269 |
| Schoenfeld Test (P-value) |  | 0.088 | 0.173 |

^#^Incidence rates per 1,000 person-years. Model 1: adjusted for age and sex. Model 2: further adjusted for education, employment, Townsend deprivation index, shift work, BMI, medical history, smoking, diet, alcohol, and statin/vitamin use. Abbreviations: MVPA, Moderate-to-Vigorous Physical Activity.

**Supplementary Table S9.** Association between PRS and risk of AAA

| **Model** | **Tertile 1** | **Tertile 2** | **Tertile 3** | ***P*_trend_** | **Schoenfeld Test (P-value)** |
| --- | --- | --- | --- | --- | --- |
| **PRS-CS** |  |  |  |  |  |
| Incident AAA^#^ | 0.163 | 0.201 | 0.503 |  |  |
| Model 1 | Ref. | 1.23 (0.82-1.84) | 3.15 (2.24-4.45) | <0.001 | 0.172 |
| Model 2 | Ref. | 1.19 (0.79-1.78) | 3.01 (2.13-4.25) | <0.001 | 0.151 |

^#^Incidence rates per 1,000 person-years. Model 1: adjusted for age and sex. Model 2: further adjusted for education, employment, Townsend deprivation index, shift work, BMI, medical history, smoking, diet, alcohol, and statin/vitamin use.

**Supplementary Table S10.** Multiplicative interactions between rest-activity rhythm and multiple variables

| **Variable** | **RA (P_interaction_)** | **M10 (P_interaction_)** | **Amplitude (P_interaction_)** | **Mesor (P_interaction_)** |
| --- | --- | --- | --- | --- |
| PRS-CS | 0.311 | 0.504 | 0.202 | 0.895 |
| **Lifestyle** |  |  |  |  |
| Smoke | 0.496 | 0.094 | 0.266 | 0.872 |
| Drink | 0.259 | 0.148 | 0.362 | 0.254 |
| Diet | 0.055 | 0.462 | 0.181 | 0.435 |
| MVPA time (Tertiles) | 0.412 | 0.358 | 0.781 | 0.426 |
| MVPA (WHO activity level classification) | 0.342 | 0.408 | 0.863 | 0.477 |
| MVPA (Weekly activity pattern) | 0.342 | 0.409 | 0.863 | 0.477 |
| LPA time (Tertiles) | 0.705 | 0.544 | 0.874 | 0.826 |
| Sedentary time (Tertiles) | 0.802 | 0.661 | 0.886 | 0.791 |
| Sleep time (Tertiles) | 0.758 | 0.696 | 0.731 | 0.763 |

All analyses were adjusted for age, sex, education, employment status, Townsend deprivation index, shift work, BMI, medical history, smoking status, diet, alcohol consumption, and statin/vitamin use, with the corresponding stratified variable excluded from adjustment in each subgroup analysis. Abbreviations: MVPA, Moderate-to-Vigorous Physical Activity.

**Supplementary Table S11.** Additive interactions between rest-activity rhythm and multiple variables

| **Variable** | **RA (P_RERI_)** | **M10 (P_RERI_)** | **Amplitude (P_RERI_)** | **Mesor (P_RERI_)** |
| --- | --- | --- | --- | --- |
| PRS-CS | 0.438 | 0.213 | 0.939 | 0.137 |
| **Lifestyle** |  |  |  |  |
| Smoke | 0.059 | 0.112 | 0.366 | 0.067 |
| Drink | 0.271 | 0.121 | 0.301 | 0.176 |
| Diet | 0.134 | 0.083 | 0.491 | 0.159 |
| MVPA time (Tertiles) | 0.339 | 0.349 | 0.702 | 0.316 |
| MVPA (WHO activity level classification) | 0.248 | 0.411 | 0.286 | 0.371 |
| MVPA (Weekly activity pattern) | 0.248 | 0.411 | 0.286 | 0.372 |
| LPA time (Tertiles) | 0.246 | 0.135 | 0.852 | 0.723 |
| Sedentary time (Tertiles) | 0.091 | 0.072 | 0.226 | 0.128 |
| Sleep time (Tertiles) | 0.675 | 0.504 | 0.129 | 0.209 |

All analyses were adjusted for age, sex, education, employment status, Townsend deprivation index, shift work, BMI, medical history, smoking status, diet, alcohol consumption, and statin/vitamin use, with the corresponding stratified variable excluded from adjustment in each subgroup analysis. Abbreviations: MVPA, Moderate-to-Vigorous Physical Activity; RERI, Relative Excess Risk due to Interaction.

**Supplementary Table S12.** Joint interactions between rest–activity rhythm and multiple variables

| **Variable** | **RA** | **M10** | **Amplitude** | **Mesor** |
| --- | --- | --- | --- | --- |
| PRS-CS | 4.74 (2.27-9.91) | 4.22 (2.09-8.49) | 4.95 (2.36-10.37) | 3.88 (1.85-8.13) |
| **Lifestyle** |  |  |  |  |
| Smoke | 7.98 (3.98-15.99) | 7.72 (3.95-15.07) | 8.33 (4.14-16.77) | 6.19 (3.06-12.52) |
| Drink | 1.81 (1.01-3.27) | 2.11 (1.13-3.95) | 1.97 (1.07-3.64) | 2.03 (1.06-3.88) |
| Diet | 3.63 (0.89-9.78) | 1.30 (0.57-2.98) | 1.94 (0.71-5.31) | 1.14 (0.49-2.63) |
| MVPA time (Tertiles) | 1.37 (0.87-2.15) | 1.20 (0.78-1.85) | 1.24 (0.81-1.90) | 1.34 (0.84-2.14) |
| MVPA (WHO activity level classification) | 1.59 (1.05-2.42) | 1.49 (0.98-2.26) | 1.52 (1.01-2.29) | 1.59 (1.01-2.48) |
| MVPA (Weekly activity pattern) | 1.71 (1.04-2.82) | 1.77 (1.05-2.99) | 1.56 (0.96-2.53) | 2.39 (1.27-4.49) |
| LPA time (Tertiles) | 1.22 (0.75-1.99) | 1.42 (0.89-2.27) | 1.50 (0.91-2.47) | 1.38 (0.86-2.22) |
| Sedentary time (Tertiles) | 1.34 (0.83-2.15) | 1.73 (1.05-2.86) | 1.83 (1.09-3.06) | 2.07 (1.17-3.67) |
| Sleep time (Tertiles) | 1.91 (1.02-3.59) | 1.70 (0.93-3.12) | 1.49 (0.82-2.72) | 1.99 (0.97-4.08) |

All analyses were adjusted for age, sex, education, employment status, Townsend deprivation index, shift work, BMI, medical history, smoking status, diet, alcohol consumption, and statin/vitamin use, with the corresponding stratified variable excluded from adjustment in each subgroup analysis. Abbreviations: MVPA, Moderate-to-Vigorous Physical Activity.

**Supplementary Table S13.** Mediating effects of inflammatory markers on the association between rest-activity rhythm parameters and the risk of AAA

| **Proportion mediated by inflammatory markers (%, 95% CI)** | **RA** | **M10** | **Amplitude** | **Mesor** |
| --- | --- | --- | --- | --- |
| Neutrophil count | 5.8 (1.2-19.4) | 5.3 (0.9-16.2) | 5.6 (1.0-18.8) | 5.1 (0.7-14.7) |
| Monocyte count | 5.3 (1.1-14.9) | 5.7 (1.1-13.1) | 6.1 (1.2-15.7) | 5.4 (1.1-12.6) |
| Lymphocyte count | 4.1 (1.1-11.4) | 4.7 (1.2-13.1) | 4.4 (0.9-13.2) | 4.8 (1.1-12.4) |
| Platelet count | NA^*^ | NA | NA | NA |
| SII score | NA | NA | NA | NA |
| SIRI score | 2.9 (0.7-7.1) | 2.8 (0.5-7.5) | 2.1 (0.8-6.9) | 1.7 (0.6-6.8) |

^*^NA indicates no significant mediation effect (p > 0.05). All analyses were adjusted for age, sex, education, employment status, Townsend deprivation index, shift work, BMI, medical history, smoking status, diet, alcohol consumption, and statin/vitamin use.

**Supplementary Table S14.** Predictive performance of four XGBoost models for abdominal aortic aneurysm

| **Model** | **Brier score** | **5-fold CV AUC** |
| --- | --- | --- |
| Characteristics | 0.002856 | 0.8474 |
| Characteristics + PRS | 0.002769 | 0.8639 |
| Characteristics + RAR | 0.002812 | 0.8508 |
| Characteristics + PRS + RAR | 0.002709 | 0.8651 |

**Characteristics** included demographic, socioeconomic, lifestyle, baseline health, medication/supplement use, and physical activity related variables. **Abbreviations:** RAR, rest-activity rhythm; PRS, polygenic risk score; AUC, area under the curve.

**Supplementary Figure S1.** Flowchart of study participants


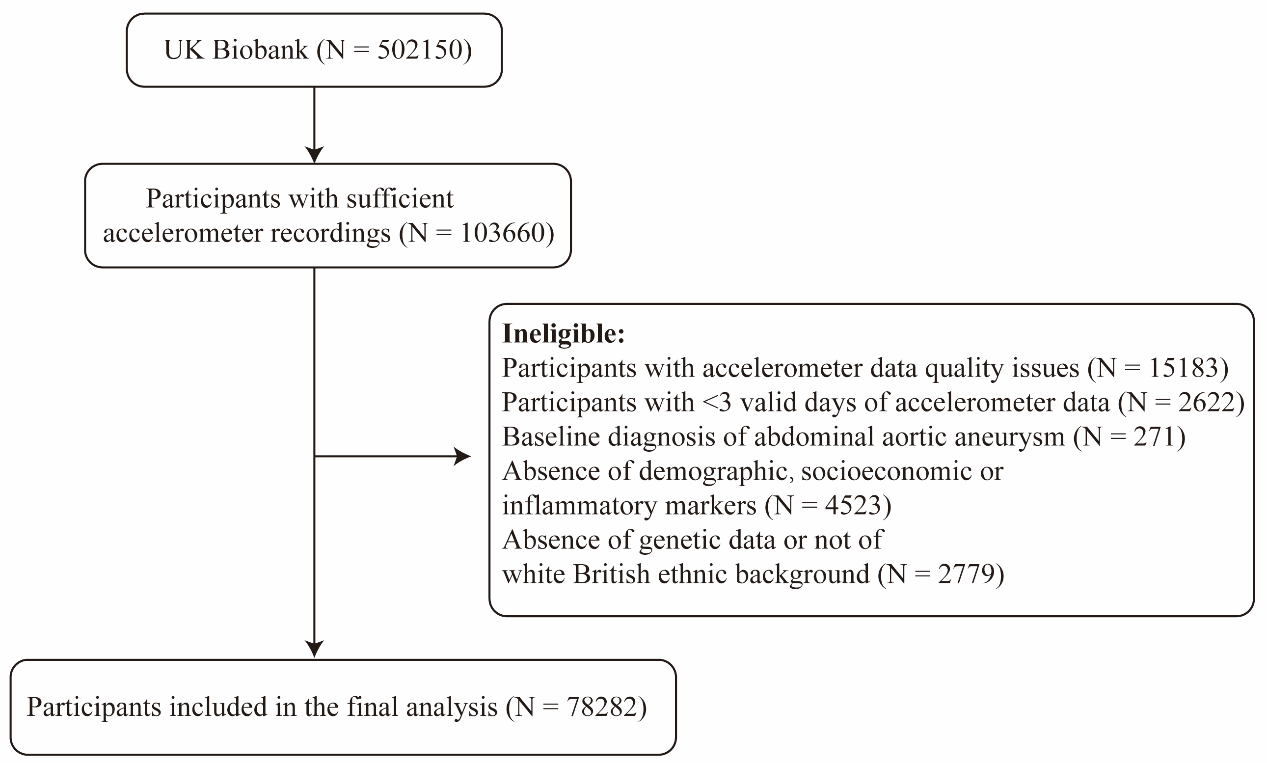


**Supplementary Figure S2.** Restricted cubic spline analyses of the associations between four rest–activity rhythm parameters and the risk of AAA


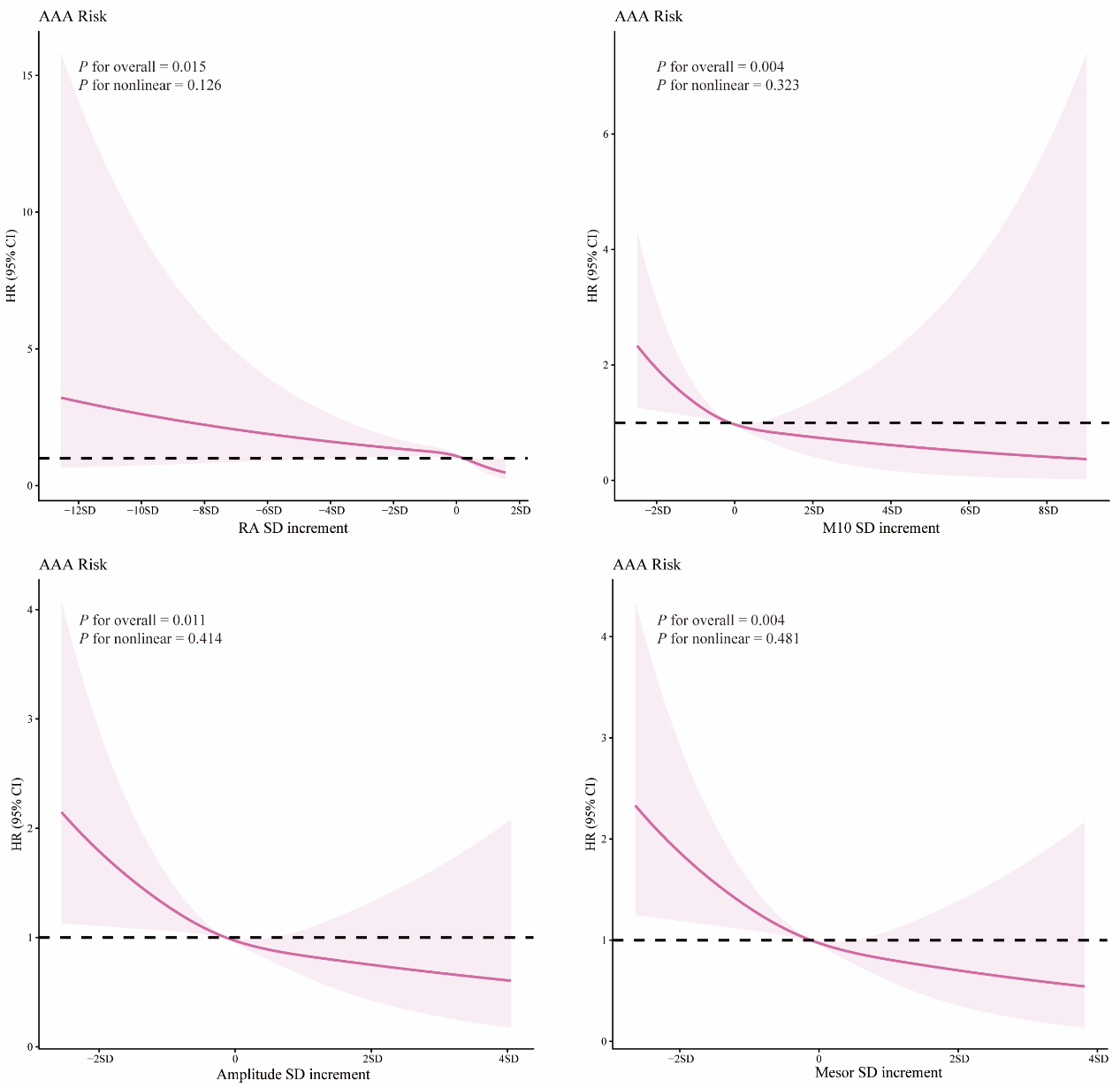


All analyses were adjusted for age, sex, education, employment status, Townsend deprivation index, shift work, BMI, medical history, smoking status, diet, alcohol consumption, and statin/vitamin use.

**Supplementary Figure S3.** ROC and decision curve analyses of four XGBoost models for AAA prediction

**
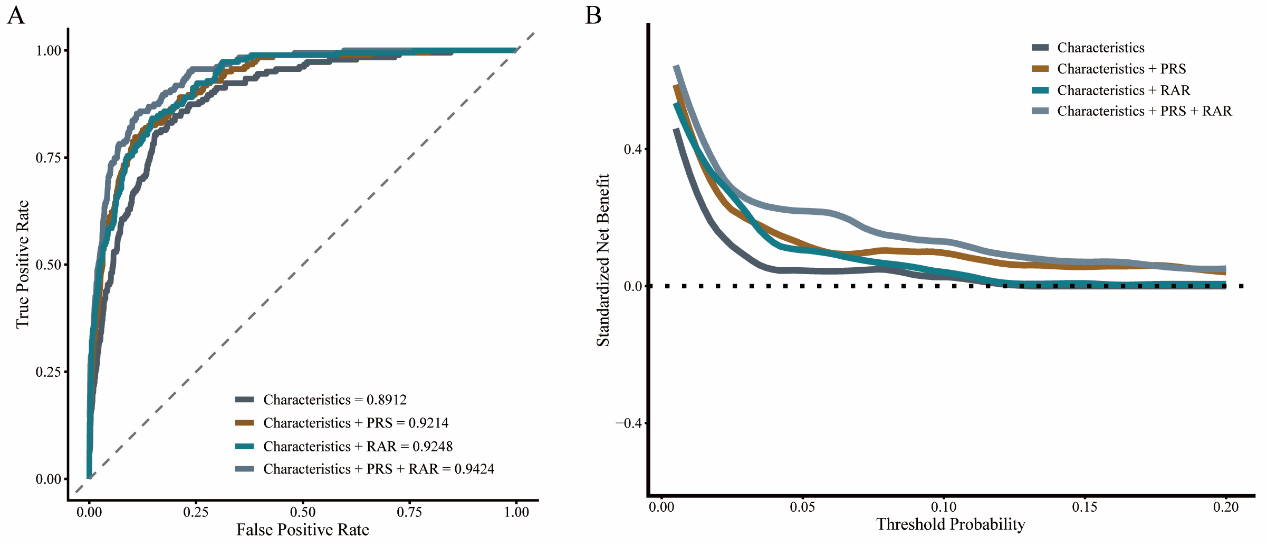
**

**(A)** ROC curves of four XGBoost models for AAA prediction. (B) Decision curve analysis of four XGBoost models for AAA prediction. **Characteristics** included demographic, socioeconomic, lifestyle, baseline health, medication/supplement use, and physical activity related variables. **Abbreviations:** RAR, rest-activity rhythm; PRS, polygenic risk score.

**Supplementary Figure S4.** Calibration plots of four XGBoost models for AAA prediction

**
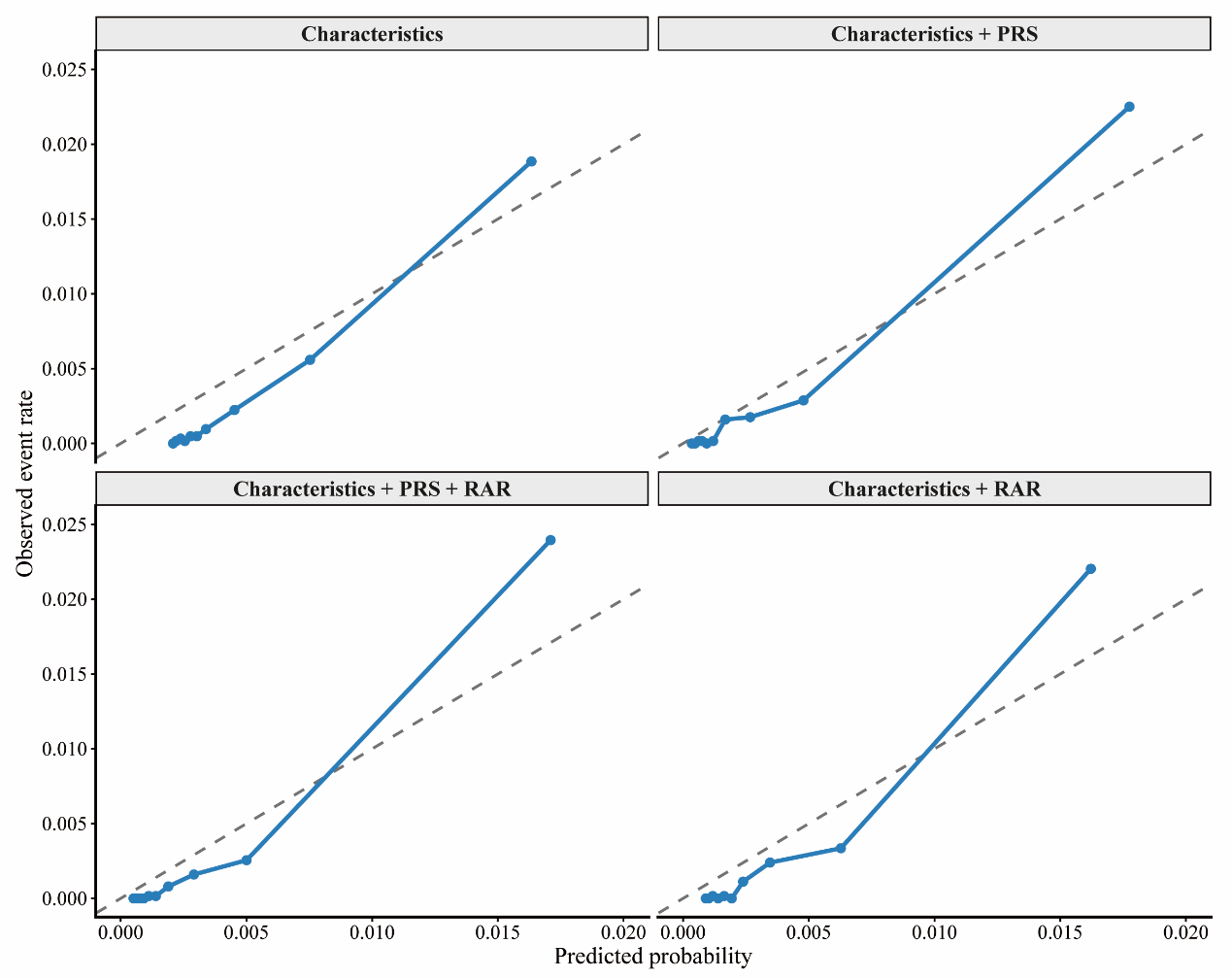
**

**Characteristics** included demographic, socioeconomic, lifestyle, baseline health, medication/supplement use, and physical activity related variables. **Abbreviations:** RAR, rest-activity rhythm; PRS, polygenic risk score.

**Supplementary Figure S5.** Feature importance ranked by mean absolute SHAP values in the XGBoost model


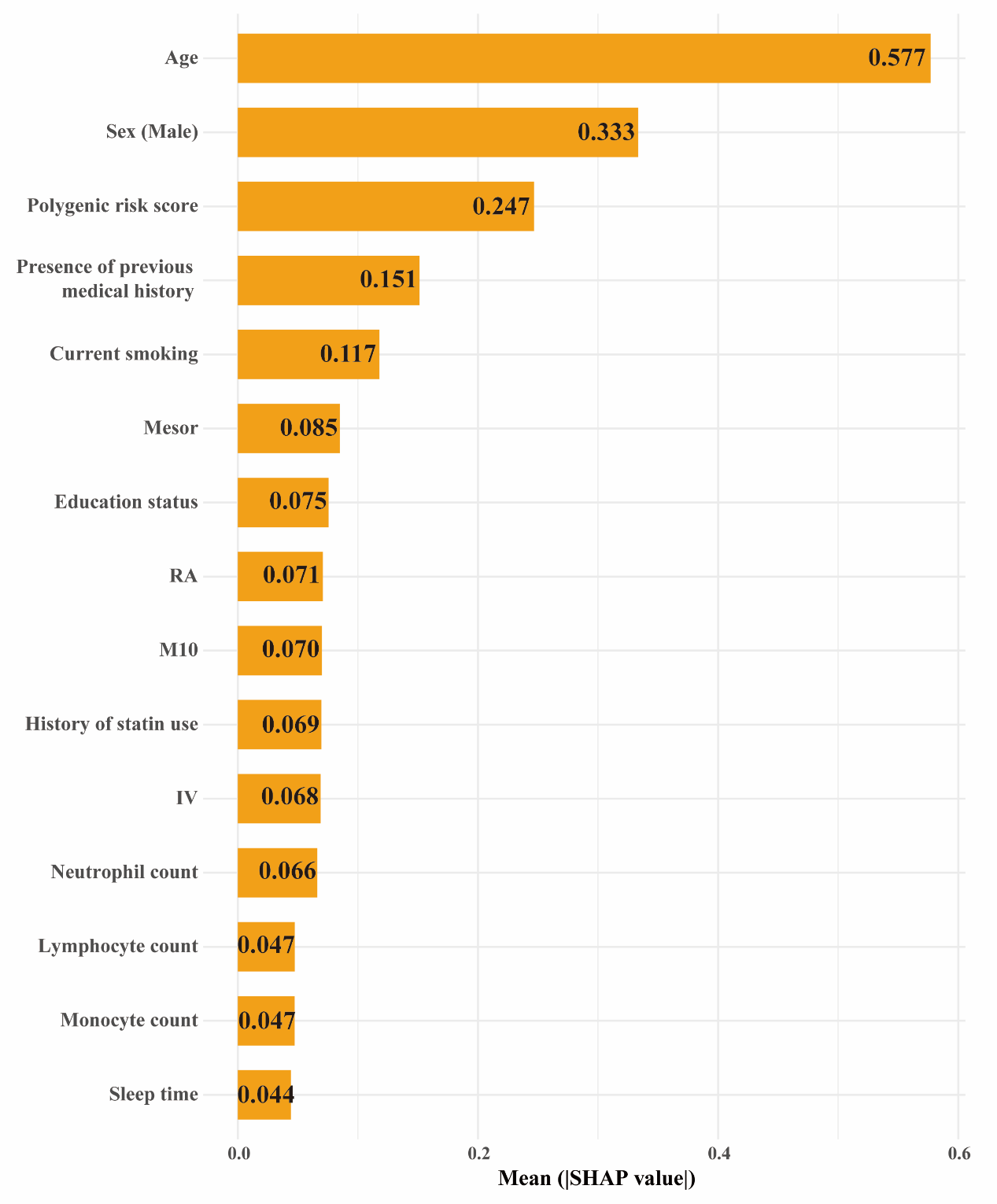


The horizontal axis represents the mean absolute SHAP value, indicating the average magnitude of each variable’s contribution to the model output (log-hazard). Variables are ranked vertically in descending order of importance.
